# Telemental Health Use in the COVID-19 Pandemic: A Scoping Review and Evidence Gap Mapping

**DOI:** 10.3389/fpsyt.2021.748069

**Published:** 2021-11-08

**Authors:** Amit Abraham, Anupama Jithesh, Sathyanarayanan Doraiswamy, Nasser Al-Khawaga, Ravinder Mamtani, Sohaila Cheema

**Affiliations:** ^1^Institute for Population Health, Weill Cornell Medicine-Qatar, Ar-Rayyan, Qatar; ^2^Weill Cornell Medicine-Qatar, Ar-Rayyan, Qatar

**Keywords:** COVID-19, coronavirus disease, telemedicine, mental health, psychiatry

## Abstract

**Background:** The COVID-19 pandemic has highlighted telemedicine use for mental illness (telemental health).

**Objective:** In the scoping review, we describe the scope and domains of telemental health during the COVID-19 pandemic from the published literature and discuss associated challenges.

**Methods:** PubMed, EMBASE, and the World Health Organization's Global COVID-19 Database were searched up to August 23, 2020 with no restrictions on study design, language, or geographical, following an *a priori* protocol (https://osf.io/4dxms/). Data were synthesized using descriptive statistics from the peer-reviewed literature and the National Quality Forum's (NQF) framework for telemental health. Sentiment analysis was also used to gauge patient and healthcare provider opinion toward telemental health.

**Results:** After screening, we identified 196 articles, predominantly from high-income countries (36.22%). Most articles were classified as commentaries (51.53%) and discussed telemental health from a management standpoint (86.22%). Conditions commonly treated with telemental health were depression, anxiety, and eating disorders. Where data were available, most articles described telemental health in a home-based setting (use of telemental health at home by patients). Overall sentiment was neutral-to-positive for the individual domains of the NQF framework.

**Conclusions:** Our findings suggest that there was a marked growth in the uptake of telemental health during the pandemic and that telemental health is effective, safe, and will remain in use for the foreseeable future. However, more needs to be done to better understand these findings. Greater investment into human and financial resources, and research should be made by governments, global funding agencies, academia, and other stakeholders, especially in low- and middle- income countries. Uniform guidelines for licensing and credentialing, payment and insurance, and standards of care need to be developed to ensure safe and optimal telemental health delivery. Telemental health education should be incorporated into health professions curricula globally. With rapidly advancing technology and increasing acceptance of interactive online platforms amongst patients and healthcare providers, telemental health can provide sustainable mental healthcare across patient populations.

**Systematic Review Registration:** https://osf.io/4dxms/.

## Introduction

Mental illness is a significant global public health issue; it is estimated to account for 13% of disability-adjusted life-years and 32.4% of years lived with disability (1). The ongoing COVID-19 pandemic has exacerbated the mental illness of vulnerable communities and individuals. Those with mental illnesses are highly vulnerable to suffer exacerbations during times of stress, such as the COVID-19 pandemic, due to their reliance on their social support network and their propensity to loneliness and isolation (2). Multiple reports indicate a rise in the prevalence of depression, anxiety, and substance use across the demographic spectrum since the onset of the COVID-19 pandemic (3–7).

During the pandemic, there was an increase in the uptake of telehealth. This digital tool, which was used sparingly prior to the pandemic, has helped enhance access to healthcare services and has proven to be safe and effective for evaluation and management (8, 9). It further leverages the expertise of highly specialized professionals across the globe (9). The myriad methods of telehealth delivery emphasize its potential to connect with marginalized populations, such as refugees or those living in remote areas (9).

Several terminologies in the published literature are currently interchangeably used to characterize telehealth related to mental health. In this review, we use the term “telemental health” [a term previously used in the literature and the National Institute of Mental Health (10–13)] in order to comprehensively describe the complete scope of Internet and Communication Technologies (ICT) based range of diagnostic and preventive mental health services. Scholarly literature has ascertained the need to guide clinical and public health decision-making by conducting scoping reviews to identify evidence, clarify concepts and characteristics, and determine research gaps (14, 15). This differentiates it from a systematic review, which is used to answer specific and well-defined research questions. As our purpose was to characterize the nature and context of the evidence in the published literature on telemental health, we decided to conduct a scoping review. The objective for this scoping review and evidence synthesis is to delineate and map the scope and domains of telemental health during the COVID-19 pandemic from the published literature and discuss associated challenges.

## Methods

### Overview

The scoping review was conducted in accordance with the Joanna Briggs Institute (JBI) Reviewer Manual (16, 17), the framework suggested by Arksey and O'Malley (15), and the evidence and gap map was based on the Campbell Collaboration Guidance (18). The protocol was registered on the Open Science Framework (registration ID: https://osf.io/4dxms) (19).The scoping review is reported using the Preferred Reporting Items for Systematic Reviews and Meta-Analyses extension for Scoping Reviews (PRISMA-ScR) (Supplementary Material 1) (20) and the PRISMA for Abstracts Checklist (Supplementary Material 2) (21).

### Eligibility Criteria

The eligibility criteria were established *a priori* as described in the protocol and are summarized here. All article types published within the COVID-19 pandemic context were included–viewpoints, observational articles, qualitative data, and systematic reviews. We considered all articles that described the use of any form of ICT, such as telephone calls, text messaging, video conferencing, smartphone applications, websites, blogs, store-and-forward, etc., for the purpose of prevention, screening, diagnosis, treatment, counseling, rehabilitation, and any other form of mental healthcare during COVID-19 as telemental health outcomes, as determined by the WHO definition of telemedicine (22, 23). We included any type of population without restriction (e.g., adults, children & adolescents, people with and without chronic disease, people with and without pre-existing mental illness, people who were diagnosed with COVID-19 etc.), provided that some ICT modality was used to provide for their mental health needs. No language or geographical restrictions were implemented.

We excluded any article that was not relevant to mental health or that focused exclusively on telehealth use in medical education or explored the use of telemental health prior to the onset of the COVID-19 pandemic.

### Search Strategy

Two reviewers (AA and SD) systematically searched Medline (via PubMed), Embase, and the World Health Organization's (WHO) Global COVID-19 Research Database, with an end-date of August 23, 2020. The end-date coincided with the increasing recognition of the mental health consequences of the pandemic and the emergence of literature to that effect (24, 25). For Medline and Embase, both controlled vocabulary and keyword searches (a combination of keywords related to COVID-19, mental health, and telehealth) were used. The WHO Global COVID-19 Research Database allows a keyword search only. Details are provided in Supplementary Material 3. We also systematically checked the bibliographies of relevant included articles for additional references. Further, we created a database of articles from related scoping reviews that we previously conducted (23, 26) and, from these, were able to identify several articles that were not picked up in our initial search strategy. The search strategy was verified by a senior librarian from Weill Cornell Medicine-Qatar.

### Study Selection

AA removed all identified duplicates using Rayyan (27), the systematic review software, following which two reviewers (AA and AJ) screened the identified articles in a two-stage screening process (title/abstract screening and full-text screening). Discrepancies at both stages were reconciled through discussion with the team. The reasons for exclusion at each step were recorded.

### Data Extraction

A standardized charting sheet was developed by AA, AJ, and SD to extract relevant information, which was modified after piloting on a small sample of articles. Once the form was finalized, AA and AJ each extracted 50% of the identified articles and then checked the remaining 50% of each other's extraction. Discrepancies were resolved between AA and AJ while keeping the review team informed. If any retrieved article was in a language unknown to the authors, the article was translated into English using Google Translate.

### Data Synthesis

Data was extracted into Microsoft Excel and synthesized using descriptive statistics. Following the recommendations of the Campbell Collaboration (18), we synthesized the evidence and used gap mapping to assess the strength of the evidence included in our scoping review. We classified mental illnesses using the Diagnostic and Statistical Manual of Mental Disorders (DSM-5) criteria (28) to understand the breadth of use of telemental health. If an article described multiple DSM-5 disorders, these were tallied separately, hence the sum totals reported below may not add up to 100%.

We used the telehealth framework developed in 2017 by the National Quality Forum (NQF) (29) to measure telehealth use for delivering healthcare. The framework uses specific domains to categorize the outcomes of telehealth including: (i) Access to Care, (ii) Financial Impact, (iii) Patient Experience, (iv) Healthcare Provider Experience, and (v) Effectiveness. In addition, using the sentiment analysis framework (30, 31), we evaluated the aforementioned domains as “celebratory” (if telehealth viewed positively by the included article authors), “contingent” (if included article authors were undecided between the pros and cons of telehealth), and “concern” (if included article authors considered the negatives to outweigh the positives of telehealth), as used in previously published literature (26, 32, 33). AA performed the sentiment analysis, with SD randomly checking 50% of the analysis to ensure integrity of the data.

## Results

**Figure 1:**
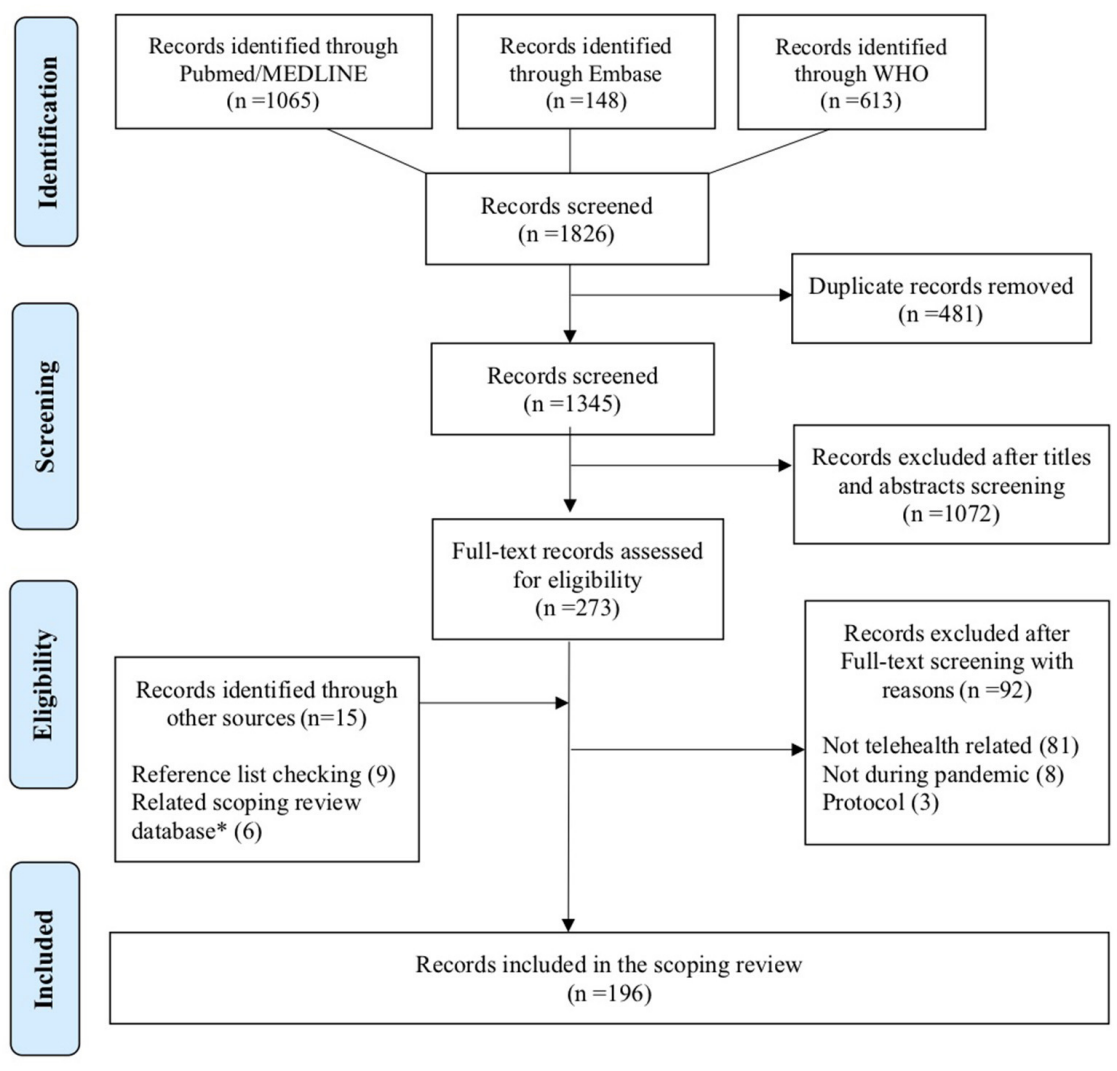
PRISMA 2009 flowchart of the systematic review's inclusion. *Database on telemedicine from previous scoping reviews.

Our primary search strategy identified 1,826 articles, from which 481 duplicates were removed. Of the remaining 1,345 articles, 1,072 were excluded during title and abstract screening and 92 during full text screening. The supplementary search strategy yielded 15 articles, so a total of 196 articles were finally included in our scoping review. This is illustrated in the PRISMA flowchart (Figure 1). The list of included articles is reported in Supplementary Material 4.

The 196 articles included in the scoping review were predominantly commentaries, viewpoints, and opinions (101, 51.53%), followed by primary studies (48; 24.45%) and reviews, recommendations, and guidelines (47; 23.98%). The articles were published across 114 journals, the majority of which were specialized in mental health, psychiatry, or related fields. The remaining journals were either general medicine journals or focused on fields, such as e-health, adolescent medicine, or occupational health. While the journal that published the highest number of the included articles followed a subscription-based model, most articles were published in hybrid or open-access journals. Most of the identified articles were published in English (194/196; 98.98%), followed by two each in French and German (2/196; 1.02%) and one each in Polish, Portuguese, and Russian (1/196; 0.51%).

The identified articles described the context of 26 countries; however, most (95/196; 48.47%) articles did not specify the country of focus. Of the articles that specified a country, most mentioned the US (31/196; 15.82%), followed by Italy and the UK, each with six articles (6/196; 3.06%). Using the WHO's regions, most articles focused on the Americas (38/196; 19.39%) and Europe (32/196; 16.32%), followed by the Western Pacific (12/196; 6.12%), South East Asia (5/196; 2.55%), and the Eastern Mediterranean (3/196; 1.53%). Most articles focused on the World Bank's high-income (71/196; 36.22%) and upper-middle income (15/196; 7.65%) countries. Only four articles focused on lower-middle income countries (4/196; 2.04%) and none on low-income countries. Detailed tables are provided in Supplementary Material 5.

The articles identified in our review were mostly written with a specific purpose of telemental health (189/196; 96.43%), while the remaining articles were general articles describing ethics of telemental health, digital privacy rights, etc. (7/196; 3.57%). Of those with a specific purpose, most of the articles described management (169/189; 89.42%); eleven articles reported both management and prevention (focused on the prevention of mental illness) (11/189; 5.82%); six articles described the preventative context only (6/189; 3.18%); two articles described both management and rehabilitative services (restoration of optimal level functioning in those with a mental illness) (2/189; 1.06%); and one article focused solely on rehabilitative services (1/189; 0.53%). The setting in which telehealth was administered was described as home- and hospital-based (55/196; 28.06%), home-based only (25/196; 12.76%), hospital-based only (14/196; 7.14%), home and school-based (2/196; 1.02%), or school-based only (1/196; 0.51%). The setting was unspecified in the remaining 99 articles (99/196; 50.51%).

There were 17 categories of healthcare providers involved in the use of telemental health in the identified articles, the most common of which were psychologists and psychiatrists (Supplementary Material 6). The specific telemental health techniques administered (such as cognitive behavioral therapy, prolonged exposure therapy, etc.) are listed in Supplementary Material 7. The telehealth modality described in the articles included telephone hotlines and telephone visits, videoconferencing software, text messaging, smartphone-based applications, online chats and emails, websites, social media, and blogs.

**Figure 2:**
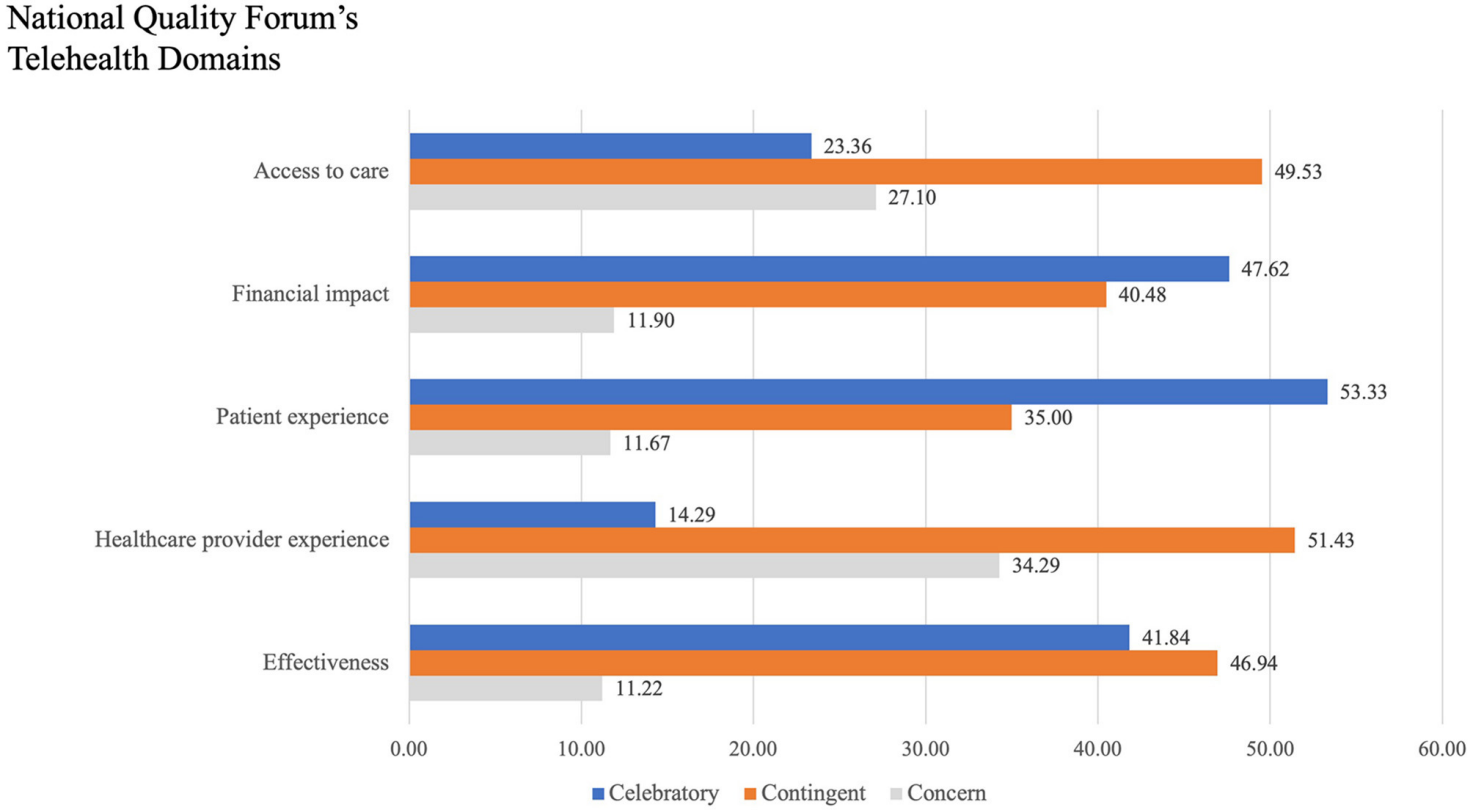
Sentiment analysis of author outlook on NQF domains.

We classified the articles using the domains of the NQF's framework (29), the results of which are depicted in Table 1. The “Access to Care” domain in the articles primarily discussed the provision of quality service to marginalized populations, technological barriers, and privacy and confidentiality issues. The “Financial Impacts” domain focused on out-of-pocket savings for individuals and families and insurance policy decisions. The “Patient Experience” domain predominantly focused on acceptability/satisfaction and the patients' home setting as both a barrier and facilitator. The principal aspects discussed under the “Healthcare Provider Experience” domain were acceptability/satisfaction, building a therapeutic alliance, and privacy and safety. With regards to the “Effectiveness” domain, the therapeutic alliance and the difference in measured outcomes were most frequently discussed. We used the sentiment analysis framework (30, 31) to describe the authors' outlook within the domains of the NQF framework, as shown in Figure 2. A majority of the included articles were “celebratory” (telehealth viewed positively by the article authors), or “contingent” (article authors were undecided between the pros and cons of telehealth), in describing the utilization of telemental health in each of the five domains. Articles classified as “concern” (article authors considered the negatives to outweigh the positives of telehealth) raised issues of privacy and confidentiality, digital equity, and technological challenges.

**Table 1:** Telemental health usage classified by the NQF's framework domains describing the objectives of included publications.

**Domains**	**Telemental health use**	
Access to care	Service people in remote locations	(34–63)
	Travel (time; efficiency; difficulty; infectious disease exposure risk)	(36, 39, 48, 50, 51, 54, 57, 58, 64–78)
	Barriers related to stigma	(35, 52, 65, 79–83)
	Transition to outpatient care	(68, 69, 76, 78, 84–87)
	Marginalized populations and healthcare disparities	(37, 41, 45, 47, 50, 53, 57, 62, 65, 70–73, 85, 88–115)
	Technology as a facilitator and barrier	(34, 37, 51, 57, 58, 60, 61, 65, 66, 72, 84, 89, 90, 92, 93, 99, 102, 104, 107, 111, 113, 116–129)
	Digital privacy and confidentiality	(43, 46, 51, 57, 60, 61, 65, 71, 74, 82, 88–90, 93, 102, 106, 108, 109, 111, 113, 117, 125, 130–136)
	Creating reserve capacity to handle increase in usage	(77)
	Licensing and regulatory issues	(39, 43, 58, 65, 89, 93, 98, 109, 113, 119, 124, 137–140)
Financial Impact	Out-of-pocket expenditure for patients and families	(34, 52, 68, 70, 74, 88, 113, 123, 134, 137, 141–144)
	Funding and costs for health service providers, hospitals, and other healthcare facilities	(71, 76, 88, 92, 95, 111, 123, 132, 144–149)
	Community cost benefits	(51, 53, 57, 83, 126, 139)
	Insurance policy on telemental health	(34, 41, 44, 47, 65, 74, 100, 102, 106, 115, 118, 130, 137, 139, 150)
Patient experience	Acceptability	(41, 52, 57, 58, 60, 69–73, 75, 76, 83, 85, 86, 92, 93, 104, 109, 110, 112, 120, 125, 127, 128, 130, 134, 145, 146, 150–159)
	Convenience	(70, 71, 86, 92, 120, 149, 156, 160, 161)
	Communication and rapport with healthcare provider	(50, 52, 91, 126, 133, 162, 163)
	Home setting as facilitator and barrier	(70, 71, 84, 89, 95, 106, 149, 163–167)
	Reduced waiting times	(42, 168)
	Safety	(72, 155, 163, 165, 167, 169)
	Privacy	(71, 92, 128, 165, 170)
Healthcare provider experience	Acceptability	(34, 47, 55, 57, 58, 60, 83, 85, 86, 92, 93, 100, 109, 119, 121, 123, 127, 128, 130, 135, 136, 138, 143, 146, 149, 152, 156, 158, 160, 163, 165, 171, 172)
	Logistics and convenience	(35, 48, 50, 52, 65, 67, 70, 71, 73, 84, 85, 95, 128, 135, 149, 168, 173)
	Communication and rapport with patients	(44, 60, 69, 70, 73, 84, 92, 93, 108, 113, 119–121, 130, 135, 138, 154, 156, 166–168, 174–177)
	Psychiatric examination and testing	(50, 60, 69–71, 111, 112, 120, 134, 156, 165, 168, 171, 174, 176, 178–180)
	Privacy and safety concerns	(34, 52, 60, 65–67, 69, 70, 92, 93, 119, 122, 126, 129, 149, 156, 163, 173, 176, 178, 181)
	Videoconferencing/“Zoom” fatigue	(52, 60, 65, 67, 161, 166, 177)
	Home setting as a facilitator and barrier	(149)
	Recorded sessions as learning for trainees	(84)
Effectiveness	Feasibility	(35, 36, 66, 68, 69, 133, 182)
	Patient engagement	(34, 58, 80, 91, 95, 122, 155, 160, 183)
	Differential outcomes (either empirical evidence or symptomatic relief)	(38, 49, 50, 53, 55, 58, 65, 68–70, 83, 85, 90, 92, 93, 100–103, 112, 114, 122, 125, 127, 130, 141, 145, 146, 151, 156, 157, 167, 170, 175, 180, 183–194)
	Therapeutic alliance and social support systems	(34, 52, 58, 59, 65, 66, 70, 71, 73, 79, 84, 89, 90, 92–95, 108–112, 116, 120–122, 127, 133, 140, 142, 143, 154, 159–161, 166, 167, 169, 176, 195–197)
	Psychiatric examination and testing	(41, 46, 50, 57, 67, 103, 107, 108, 121, 122, 127, 135, 169, 194)
	No-show rate, loss to follow-up, and medication & treatment adherence	(34, 38, 51, 70, 90, 99, 101, 134, 142, 144, 156)
	Harmful self-perception and safety	(42, 65, 122, 143, 157, 176, 198)
	Administrative workflow	(138, 141)
	Cultural considerations	(66, 71, 142)

Just over half of all articles described a specific mental health diagnosis (109/196; 55.61%), as described by the DSM-5. More specifically, the most frequently cited DSM-5 disorders were trauma and stressor-related disorders (14/196; 7.14%), anxiety disorders (13/196; 6.63%), and depressive disorders (12/196; 6.12%). The most frequent disorder subtypes, when specified, were depression (12/196; 6.12%), anxiety (10/196; 5.10%), and eating disorders (9/196; 4.59%). A total of 52 articles examined mental health disorders in specific scenarios that could not be classified using the DSM-5 criteria (such as mental health in pediatric, oncology, geriatric, and refugee-care settings), and these were grouped together under “Specialized psychiatry” (52/196; 26.53%). Table 2 illustrates the DSM-5 disorders and subtypes identified in the articles.

**Table 2:** Telemental health usage classified by DSM-5 criteria (conditions and subtypes).

**Mental disorders**	**Sub-types**	**Number of studies^**a**^**
**Anxiety disorders**		**13**
	Anxiety	10
	Generalized anxiety disorder	1
	Social anxiety disorder	1
	Unspecified	1
**Bipolar and related disorders**		**5**
	Bipolar disease	5
**Depressive disorders**		**12**
	Major depressive disorder	12
**Disruptive, impulse control, and conduct disorders**		**1**
	Unspecified	1
**Feeding and eating disorders**		**10**
	Anorexia nervosa	1
	Bulimia nervosa	1
	Binge eating disorder	1
	Unspecified	9
**Neurocognitive disorders**		**5**
	Major and mild neurocognitive disorders [traumatic brain injury]	1
	Unspecified	4
**Neurodevelopmental disorders**		**6**
	Attention-Deficit/ hyperactivity disorder	1
	Autism spectrum disorder	4
	Intellectual disability	1
	Unspecified	1
**Obsessive-Compulsive and related disorders**		**2**
	Obsessive compulsive disorders	2
**Schizophrenia spectrum and other psychotic disorders**		**7**
	Schizoaffective disorder	1
	Schizophrenia	4
	Unspecified	2
**Sleep wake disorders**		**1**
	Insomnia disorder	1
**Somatic symptom and related disorders**		**1**
	Unspecified	1
**Specialized psychiatry**		**52**
	Dermatology	1
	Ethics	1
	Immigrant, refugee, and asylum seekers	2
	Geriatrics	10
	Oncology	3
	Pediatrics	13
	Preventive medicine	2
	Psychological stress	10
	Rural mental health	1
	Serious mental illness	1
	Social isolation	3
	Suicide	1
	Unspecified	4
**Substance-Related and addictive disorders**		**8**
	Alcohol-Related disorders	1
	Cannabis-Related disorders	1
	Opioid-Related disorders	3
	Unspecified	4
**Trauma and stressor-related disorder**		**14**
	Persistent complex bereavement disorder	1
	Post-traumatic stress disorder	7
	Unspecified	6
**General psychiatric care**		**88**

*^a^Several articles described multiple DSM-5 disorders, which were tallied separately. Hence the reported totals do not necessarily add up to 100%*.

**Figure 3:**
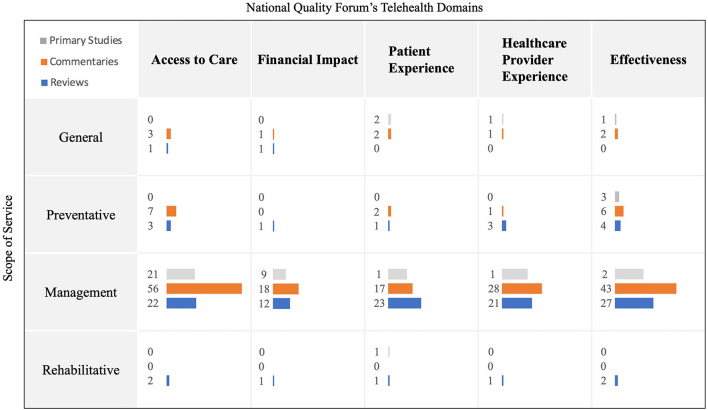
Visual map for scope of service, NQF framework and article type. Preventative: Screening/Triage/Lifestyle/Social connectedness; Management: Treatment/Follow-up Rehabilitative: restoration of optimal level functioning in those with a mental illness; General: No definitive focus; Gray bars: Primary studies; Orange bars: Commentaries, viewpoints, editorials, perspectives; Blue bars: Literature and Scoping reviews; The length of the bars and the numbers correspond to the total number of identified articles.

To inform the context of telemental health, we harmonized the evidence into a visual map by comparing the scope of service (management, preventative, rehabilitative, general) to each domain within the NQF's telemental health framework, and the design of the study (Figure 3). The map demonstrates that most articles examined management using telemental health and a few articles examined prevention or rehabilitation. Furthermore, commentaries were the most common study design included in our scoping review, nearly double of the other designs. Most articles described the Access to Care and Effectiveness domains, with relatively few focusing on the Financial Impact.

Table 3 summarizes the recommendations, where they were made, for enhancing telemental health from the included articles by NQF domain (36/196; 18.37%). Most articles called for standardizing licensing and regulatory policies, ensuring appropriate privacy and confidentiality measures, adequate preparation of patients and healthcare providers for the telemental health experience, having a back-up plan for technological glitches, and ensuring the presence of safety measures in case of a psychiatric emergency.

**Table 3:** Recommendations to enhance telemental health, classified by the NQF framework domains.

**Domains**	**Recommendations regarding telemental health use**	
Access to care	Health service providers and policy makers must both recognize and advocate to reduce health disparities	(35, 103, 126, 154)
	Ensure adequate privacy and confidentiality protections	(50, 57, 70, 72, 79, 92, 94, 95, 159, 175, 180)
	Prepare a device inventory (e.g., tablets) to share with marginalized populations	(180)
	Standardize policies on licensing and regulatory issues	(50, 53, 74, 85, 121, 126, 159, 175, 199)
Financial impact	Ensure patients are aware of billing and insurance policies up front	(72, 74)
	Ensure insurance providers expand coverage for telemental health	(53)
	Malpractice insurance for telemental health services	(175)
Patient experience	Prepare for the telemental health experience	(53, 85, 121, 126, 133, 167, 180)
	Telemental health sessions should last for reasonable lengths of time, with a periodic break, if needed	(176)
	Empower patients to participate and be an equal partner in their own care	(176)
	Have one-on-one sessions prior to group sessions, schedule the group sessions at convenient times for all using differing modalities to maximize participation	(57, 91)
Healthcare provider experience	Ensure staff receive appropriate training and practice	(42, 53, 74, 83, 93, 100, 126, 200)
	Adopt empathetic and personalized communications style and properly consent patients	(50, 57, 70, 72, 121, 140, 160)
	Provide multiple telemental health modalities and have back-ups in case of technological issues	(42, 50, 70, 72, 74, 92, 100, 195)
	Address provider and patient concerns and encourage sharing of success and challenges for quality assessment purposes	(57, 83, 159)
	Develop safety and contingency plans in conjunction with patients	(42, 72, 92, 93, 180)
Effectiveness	Enhance therapeutic alliance and dissemination of information	(94, 127, 134)
	Provide integrated care using dedicated teams	(100, 134, 201)
	Adapt appropriate cultural means of communications	(38, 49, 50, 53, 55, 58, 65, 68–70, 72, 83, 85, 90, 92, 93, 100–103, 112, 114, 122, 125–127, 130, 141, 145, 146, 151, 154, 156, 157, 167, 170, 175, 180, 183–194)
	Develop tools to enhance acceptability and safety	(134, 161)

## Discussion

In this scoping review and evidence synthesis, we collated the extent and use of telemental health during the COVID-19 pandemic and lessons learned that could be beneficial for the future. Most articles in the scoping review described the management aspects of telemental health provision, with only a few describing preventative or rehabilitative aspects. Thus, there is much scope for improvement in order to use telemental health for prevention and early diagnosis of mental illnesses. This can be facilitated by building resilience through social network and connections, augmenting social services and surveillance systems, and enhancing surge capacity and redundancy in the mental healthcare system (5). Approximately half of the articles in our review described general telemental health care rather than focusing on a specific mental health disorder. This finding illustrates the generalizability of telemental health and its application to all aspects of the telemental health continuum, from prevention to management to rehabilitation.

Our findings demonstrate that most of the identified articles described telemental health in the context of high-income countries, which is reflected in the scholarly literature (23, 26). Current data suggest that telemental health can be effectively used in these countries (23, 26). However, there is a scarcity of evidence supporting its use in low-resource settings (202–204). This highlights the importance of data collection from lower middle-income as well as low-income countries.

While telemental health is increasingly becoming more mainstream in high-income countries, the identified articles note that challenges regarding regulations, credentialing and licensing, and standards of care must be overcome. A careful cost-benefit analysis must be conducted for each practice, due to vagaries that are region-specific. Further work must be undertaken to enhance access to and affordability of technology (123). Identified data, while limited in low and middle-income countries, suggests that telemental health is beneficial in these settings. The effectiveness of telemental health may vary in more culturally diverse settings, and the cost of implementation in low- and middle-income countries is unclear. Issues surrounding privacy and confidentiality must be addressed in settings with less-than-robust healthcare systems and high levels of stigma toward mental health problems (202). A total of four identified articles discussed the ethical implications of telemental health (88, 89, 173, 178). The same ethical obligations of patient beneficence; fidelity and responsibility; distributive justice; integrity; privacy; and autonomy apply to telemental health (178, 205). However, telemental health also has unique challenges that must be considered, such as handling patient encounters occurring via third-party videoconferencing software (178, 206). Frameworks have been described in the published literature to ensure that the standards of care are adhered to, and patient autonomy and privacy are maintained (207, 208).

An important consideration in the application of telemental health is the setting in which it is used, as different NQF domains may be of varying importance in different circumstances, as highlighted in the identified articles. For instance, the privacy and security of individuals using telemental health is especially important in a school-based setting or for those being treated for substance use. The identified articles illustrated the use of telemental health across settings such as homes, hospitals, and schools. However, the majority described the use of telemental health for the provision of home-based care. This can provide the healthcare provider with insight into social and environmental conditions at home (204, 209). The healthcare provider is thus better able to assess some of the social determinants of health, information on which has been otherwise been lacking in the current modern medical system (210). Several articles also emphasized the importance of schools as an additional avenue to ensure that children have access to healthcare. Over a quarter of the identified articles discussed telemental health use in specialized situations, such as pediatrics and adolescent care, geriatrics, oncology, and refugee-care, strengthening the argument that telemental health needs to be tailored to specific scenarios in order to deliver optimal care (160).

The articles included in our review substantiate the claim that telemental health is useful in reducing barriers to mental healthcare access for traditionally marginalized communities, such as those living in remote locations, migrants, refugees, and asylum seekers. Because of the near-ubiquity of smartphones and other internet-enabled devices, telemental health can cater to the needs of such populations (211). However, challenges associated with telemental health exist in these communities that must be addressed. For instance, there is a higher likelihood of digital inequity among the vulnerable populations, with lack of affordability or access to broadband services, patients' unfamiliarity, or the inability to use ICT. In addition, a lack of privacy and higher levels of stigma may also exist as compared to other populations (212–214).

When we categorized the concerns raised in the included articles by DSM-5 criteria (28), we found depression, anxiety, and eating disorders to be the most common concerns. This is not unexpected, given concern over the severity and transmissibility of COVID-19, limited hospital capacity during the initial wave of the pandemic, misinformation and rumors about the pandemic, and the public health measures that were implemented (movement restrictions, limiting in-person interactions, quarantine, isolation, etc.) to reduce the risk of transmission (214, 215).

The NQF framework (29) for tele-mental health allows us to structure our findings. In doing so, we identified that the NQF domains that were addressed in most detail were Access to Care and Effectiveness. Most articles identified telemental health as a boon toward these domains by alleviating the stigma, and time and privacy barriers facing patients and healthcare providers. However, several articles noted challenges in using telemental health services, including the inability of certain at-risk communities to comprehend and use the technology, perceived inefficacy, and technological challenges. Overall sentiment analysis demonstrates a celebratory sentiment toward the Patient Experience and Financial Impact domains, and contingent sentiment toward the other domains. Despite a robust evidence base and the global pivot to telemental health use during the COVID-19 pandemic, there remains work to be done to ensure that it is affordable, effective, and accessible to all communities worldwide.

Another finding is that telemental health facilitates and enhances the delivery of integrated and quality care when multiple specialist healthcare providers may be needed or when the providers are remotely located (210). Specific competencies have been developed to ensure that healthcare providers are proficient in the delivery of interdisciplinary telemental health care (216, 217). Telemental health also permits family members to be involved in providing support to patients if they are in different geographic locations (210).

Several findings of this scoping review resonate with the findings in literature that has been published recently–telemental health reduced no-show rates (209), had equivalent outcomes to in-person sessions (218) and yielded satisfaction among both patients and healthcare providers (209). Challenges cited included concerns about confidentiality and privacy issues, and disparities in digital equity (218, 219). Other issues that have more recently been highlighted include screen fatigue (150, 220, 221), and a loss in sense of community for patients, due to fewer in-person interactions with fellow patients and healthcare providers (34).

Telemental health use is likely to continue to expand in the future. It confers myriad advantages, such as enhanced access to marginalized communities with limited mental health resources, mitigating individual stigma and creating reserve health system capacity. Telemental health can serve to reduce health disparities and lower the impact of future crises. The findings can help inform policymakers and healthcare institutions on making decisions about the future applications of telemental health.

Our scoping review has some limitations. We did not formally assess the quality of the included articles, the majority of which were commentaries. However, all articles were peer-reviewed, which reassures concerns about quality. Since a majority of the articles described the high-income context, the generalizability of the findings of this scoping review will likely be limited to high-income countries only. Further research in the low- and middle-income countries is warranted, and investment, perhaps with funding from high-income countries/global funding agencies, can help with the implementation of telemental health in countries where it is currently lacking.

Issues that remain unexplored in the published literature include the need for higher quality evidence, such as randomized controlled trials to ensure best clinical outcomes; best measures to address the digital divide; distinction in usage between the various telemental health modalities (videoconferencing vs. audio-only healthcare vs. text messaging systems), and best practices for training healthcare providers to transition to telemental health. We did not investigate the use of artificial intelligence, an important research topic as it is becoming a part of telemental services, as this was beyond the scope of the study. Policy makers and researchers must prioritize optimizing telemental health services to cater to at-risk populations and address the aforementioned concerns and challenges.

## Conclusion

Our findings suggest that telemental health could prove to be useful and effective during and beyond the COVID-19 pandemic. However, we must continue to explore opportunities to improve and enhance the delivery of telemental health for optimum health benefit to communities worldwide. The development of high-speed internet infrastructure globally will facilitate the uptake of telemental health. Given the dearth of comprehensive data on the outcomes of telemental health in low resource settings, as well as rural and remote communities, a greater investment into resources and additional research are needed. Guidelines and policies for licensing, geographical coverage, payment, insurance, and standard of care need to be put in place. Further, telemental health education should be incorporated into medical and health professions curricula worldwide to allow for better acceptance and familiarity among healthcare providers. Telemental health has the potential to be beneficial, especially for marginalized communities. Healthcare providers should embrace and offer evidence-based telemental health services to populations most in need in order to promote optimum health.

## Data Availability Statement

The original contributions presented in the study are included in the article/Supplementary Material, further inquiries can be directed to the corresponding author.

## Author Contributions

AA, SD, SC, and RM collectively contributed to the conception and design of the study. NA-K reviewed the literature. AA and SD designed the search strategy, while screening and data extraction were conducted by AA and AJ in consultation with SD, SC, and RM. Analysis and manuscript drafting was carried out by AA with support from SD, AJ, NA-K, SC, and RM. All authors participated in the interpretation of the results, reviewed, edited, and approved the final version of the manuscript.

## Conflict of Interest

The authors declare that the research was conducted in the absence of any commercial or financial relationships that could be construed as a potential conflict of interest.

## Publisher's Note

All claims expressed in this article are solely those of the authors and do not necessarily represent those of their affiliated organizations, or those of the publisher, the editors and the reviewers. Any product that may be evaluated in this article, or claim that may be made by its manufacturer, is not guaranteed or endorsed by the publisher.

## Abbreviations

ICT: Internet and communication technologies
JBI: Joanna briggs institute reviewer manual
NQF: national quality forum
PRISMA-ScR: Preferred reporting items for systematic reviews and meta-analyses extension for scoping reviews
WHO: World health organization.
