# Supplementary material for: Telemental Health Use in the COVID-19 Pandemic: A Scoping Review and Evidence Gap Mapping
Source: Front Psychiatry. 2021 Nov 8;12:748069. doi: 10.3389/fpsyt.2021.748069 (PMC8606591; doi:10.3389/fpsyt.2021.748069)
Supplement: Supplementary file 1 [file Data_Sheet_1.docx]

# Supplementary Material

## Supplementary Material 1: Preferred Reporting Items for Systematic reviews and Meta-Analyses extension for Scoping Reviews (PRISMA-ScR) Checklist

| **SECTION** | **ITEM** | **PRISMA-ScR CHECKLIST ITEM** | **REPORTED ON PAGE #** |
| --- | --- | --- | --- |
| **TITLE** | | | |
| Title | 1 | Identify the report as a scoping review. | 1 |
| **ABSTRACT** | | | |
| Structured summary | 2 | Provide a structured summary that includes (as applicable): background, objectives, eligibility criteria, sources of evidence, charting methods, results, and conclusions that relate to the review questions and objectives. | 1 |
| **INTRODUCTION** | | | |
| Rationale | 3 | Describe the rationale for the review in the context of what is already known. Explain why the review questions/objectives lend themselves to a scoping review approach. | 1 |
| Objectives | 4 | Provide an explicit statement of the questions and objectives being addressed with reference to their key elements (e.g., population or participants, concepts, and context) or other relevant key elements used to conceptualize the review questions and/or objectives. | 1 |
| **METHODS** | | | |
| Protocol and registration | 5 | Indicate whether a review protocol exists; state if and where it can be accessed (e.g., a Web address); and if available, provide registration information, including the registration number. | 2 |
| Eligibility criteria | 6 | Specify characteristics of the sources of evidence used as eligibility criteria (e.g., years considered, language, and publication status), and provide a rationale. | 2 |
| Information sources* | 7 | Describe all information sources in the search (e.g., databases with dates of coverage and contact with authors to identify additional sources), as well as the date the most recent search was executed. | 3 |
| Search | 8 | Present the full electronic search strategy for at least 1 database, including any limits used, such that it could be repeated. | 3 |
| Selection of sources of evidence† | 9 | State the process for selecting sources of evidence (i.e., screening and eligibility) included in the scoping review. | 3 |
| Data charting process‡ | 10 | Describe the methods of charting data from the included sources of evidence (e.g., calibrated forms or forms that have been tested by the team before their use, and whether data charting was done independently or in duplicate) and any processes for obtaining and confirming data from investigators. | 3 |
| Data items | 11 | List and define all variables for which data were sought and any assumptions and simplifications made. | 3 |
| Critical appraisal of individual sources of evidence§ | 12 | If done, provide a rationale for conducting a critical appraisal of included sources of evidence; describe the methods used and how this information was used in any data synthesis (if appropriate). | N/A |
| Synthesis of results | 13 | Describe the methods of handling and summarizing the data that were charted. | 3-4 |
| **RESULTS** | | | |
| Selection of sources of evidence | 14 | Give numbers of sources of evidence screened, assessed for eligibility, and included in the review, with reasons for exclusions at each stage, ideally using a flow diagram. | 4, Figure 1 |
| Characteristics of sources of evidence | 15 | For each source of evidence, present characteristics for which data were charted and provide the citations. | 4-7 |
| Critical appraisal within sources of evidence | 16 | If done, present data on critical appraisal of included sources of evidence (see item 12). | N/A |
| Results of individual sources of evidence | 17 | For each included source of evidence, present the relevant data that were charted that relate to the review questions and objectives. | 4-7 |
| Synthesis of results | 18 | Summarize and/or present the charting results as they relate to the review questions and objectives. | 4-7 |
| **DISCUSSION** | | | |
| Summary of evidence | 19 | Summarize the main results (including an overview of concepts, themes, and types of evidence available), link to the review questions and objectives, and consider the relevance to key groups. | 7-9 |
| Limitations | 20 | Discuss the limitations of the scoping review process. | 8 |
| Conclusions | 21 | Provide a general interpretation of the results with respect to the review questions and objectives, as well as potential implications and/or next steps. | 8-9 |
| **FUNDING** | | | |
| Funding | 22 | Describe sources of funding for the included sources of evidence, as well as sources of funding for the scoping review. Describe the role of the funders of the scoping review. | 9 |

JBI = Joanna Briggs Institute; PRISMA-ScR = Preferred Reporting Items for Systematic reviews and Meta-Analyses extension for Scoping Reviews.

* Where *sources of evidence* (see second footnote) are compiled from, such as bibliographic databases, social media platforms, and Web sites.

† A more inclusive/heterogeneous term used to account for the different types of evidence or data sources (e.g., quantitative and/or qualitative research, expert opinion, and policy documents) that may be eligible in a scoping review as opposed to only studies. This is not to be confused with *information sources* (see first footnote).

‡ The frameworks by Arksey and O’Malley (6) and Levac and colleagues (7) and the JBI guidance (4, 5) refer to the process of data extraction in a scoping review as data charting*.*

§ The process of systematically examining research evidence to assess its validity, results, and relevance before using it to inform a decision. This term is used for items 12 and 19 instead of "risk of bias" (which is more applicable to systematic reviews of interventions) to include and acknowledge the various sources of evidence that may be used in a scoping review (e.g., quantitative and/or qualitative research, expert opinion, and policy document).

*From:* Tricco AC, Lillie E, Zarin W, O'Brien KK, Colquhoun H, Levac D, et al. PRISMA Extension for Scoping Reviews (PRISMAScR): Checklist and Explanation. Ann Intern Med. 2018;169:467–473. [doi: 10.7326/M18-0850](http://annals.org/aim/fullarticle/2700389/prisma-extension-scoping-reviews-prisma-scr-checklist-explanation).

## Supplementary Material 2: PRISMA for Abstracts Checklist

| **TITLE** | **CHECKLIST ITEM** | REPORTED ON PAGE # |
| --- | --- | --- |
| 1. Title: | Identify the report as a systematic review, meta-analysis, or both. | 1 |
| **BACKGROUND** |  |  |
| 2. Objectives: | The research question including components such as participants, interventions, comparators, and outcomes. | 1 |
| **METHODS** |  |  |
| 3. Eligibility criteria: | Study and report characteristics used as criteria for inclusion. | 1 |
| 4. Information sources: | Key databases searched and search dates. | 1 |
| 5. Risk of bias: | Methods of assessing risk of bias. | N/A |
| **RESULTS** |  |  |
| 6. Included studies: | Number and type of included studies and participants and relevant characteristics of studies. | 1 |
| 7. Synthesis of results: | Results for main outcomes (benefits and harms), preferably indicating the number of studies and participants for each. If meta-analysis was done, include summary measures and confidence intervals. | 1 |
| 8. Description of the effect: | Direction of the effect (i.e. which group is favoured) and size of the effect in terms meaningful to clinicians and patients. | N/A |
| **DISCUSSION** |  |  |
| 9. Strengths and Limitations of evidence: | Brief summary of strengths and limitations of evidence (e.g. inconsistency, imprecision, indirectness, or risk of bias, other supporting or conflicting evidence) | 1 |
| 10. Interpretation: | General interpretation of the results and important implications | 1 |
| **OTHER** |  |  |
| 11. Funding: | Primary source of funding for the review. | 1 |
| 12. Registration: | Registration number and registry name. | 1 |

## Supplementary Material3: Search Strategy

### PubMed

| **Search number** | **Query** | **Results** | **Date** |
| --- | --- | --- | --- |
|  |  |  |  |
| 1 | (telemedicine[MeSH Terms]) OR (telehealth[MeSH Terms]) OR mobile health[MeSH Terms] | 29,317 | Aug 23 2020 |
| 2 | "tele*"[All Fields] OR "digital*"[All Fields] OR "remote*"[All Fields] OR "video*"[All Fields] OR "Ehealth"[All Fields] OR "e-health"[All Fields] OR "e-health"[All Fields] OR "electronic health"[All Fields] OR "virtual"[All Fields] OR "virtuality"[All Fields] OR "virtualization"[All Fields] OR "virtualized"[All Fields] OR "virtualizing"[All Fields] OR "virtuals"[All Fields] | 712,635 | Aug 23 2020 |
| 3 | #1 or #2 | 712,635 | Aug 23 2020 |
| 4 | "Psychiatry"[Mesh] or ("Counseling"[Mesh]) OR "Mental Health Services"[Mesh] | 194,838 | Aug 23 2020 |
| 5 | psychiatr* or psycholo* or "mental health" or anxiety or panic or agora* or phobia or OCD or obsess* or compuls* or Bipolar or "conduct disorder" or "eating disorder" or anorexia or bulimia or "mood disorder" or depress* or dysthymia or affect or psycho* or neurotic or paraphilia or schizo* or autism or ADHD or trauma or PTSD or stress OR substance abuse OR "recreational drug" or "personality disorder" or counsel* OR CBT or psychotherapy or "behavioral therapy" or ECT or "electroconvulsive therapy" or psychoanalysis | 6,587,658 | Aug 23 2020 |
| 6 | #4 or #5 | 6,607,137 | Aug 23 2020 |
| 7 | (((((("covid 19"[All Fields] OR "covid 2019"[All Fields]) OR "severe acute respiratory syndrome coronavirus 2"[Supplementary Concept]) OR "severe acute respiratory syndrome coronavirus 2"[All Fields]) OR "2019 ncov"[All Fields]) OR "sars cov 2"[All Fields]) OR "2019ncov"[All Fields]) OR (("wuhan"[All Fields] AND ("coronavirus"[MeSH Terms] OR "coronavirus"[All Fields])) AND (2019/12/1:2019/12/31[Date - Publication] OR 2020/1/1:2020/12/31[Date - Publication])) | 42,751 | Aug 23 2020 |
| 8 | #3 and #6 and #7 | 1,065 | Aug 23 2020 |

### EMBASE

| **Search number** | **Query** | **Results** | **Date** |
| --- | --- | --- | --- |
|  |  |  |  |
| 1 | exp social psychiatry/ or exp cultural psychiatry/ or exp child psychiatry/ or psychiatry.mp. or exp emergency psychiatry/ or exp psychiatry/ | 162,123 | Aug 23 2020 |
| 2 | exp patient counseling/ or counseling.mp. or exp counseling/ or exp family counseling/ or exp e-counseling/ or exp directive counseling/ | 193,455 | Aug 23 2020 |
| 3 | (psychiatr* or psycholo* or "mental health" or anxiety or panic or agora* or phobia or OCD or obsess* or compuls* or Bipolar or "conduct disorder" or "eating disorder" or anorexia or bulimia or "mood disorder" or depress* or dysthymia or affect or psycho* or neurotic or paraphilia or schizo* or autism or ADHD or trauma or PTSD or stress OR substance abuse OR "recreational drug" or "personality disorder" or counsel* OR CBT or psychotherapy or "behavioral therapy" or ECT or "electroconvulsive therapy" or psychoanalysis).mp. [mp=title, abstract, heading word, drug trade name, original title, device manufacturer, drug manufacturer, device trade name, keyword, floating subheading word, candidate term word] | 4,789,443 | Aug 23 2020 |
| 4 | 1 or 2 or 3 | 4,796,071 | Aug 23 2020 |
| 5 | telehealth.mp. or exp telehealth/ | 48,377 | Aug 23 2020 |
| 6 | telemedicine.mp. or exp telemedicine/ | 45,482 | Aug 23 2020 |
| 7 | (tele* or digital* or remote or video* or Ehealth or e-health or "electronic health" or virtual).mp. [mp=title, abstract, heading word, drug trade name, original title, device manufacturer, drug manufacturer, device trade name, keyword, floating subheading word, candidate term word] | 820,429 | Aug 23 2020 |
| 8 | 5 or 6 or 7 | 820,429 | Aug 23 2020 |
| 9 | 4 and 8 | 146,723 | Aug 23 2020 |
| 10 | limit 9 to covid-19 | 972 | Aug 23 2020 |
| 11 | limit 10 to exclude medline journals | 148 | Aug 23 2020 |

### WHO Global COVID-19 database

| **Search number** | **Query** | **Results** | **Date** |
| --- | --- | --- | --- |
|  |  |  |  |
| 1 | (tw:(psychiatr* or psycholog* or "mental health" or anxiety or panic or agora* or phobia or OCD or Bipolar or "conduct disorder" or "eating disorder" or anorexia or bulimia or "mood disorder" or depress* or dysthymia or affect or psycho* or neurotic or paraphilia or schizo* or autism or ADHD or trauma or PTSD or stress OR substance abuse OR "recreational drug" or "personality disorder" or counsel* OR CBT or psychotherapy or "behavioral therapy" or ECT or "electroconvulsive therapy" or psychoanalysis)) | 6720 | Aug 23 2020 |
| 2 | (tw:("tele*"[All Fields] OR "digital*"[All Fields] OR "remote*"[All Fields] OR "video*"[All Fields] OR "Ehealth"[All Fields] OR "e-health"[All Fields] OR "e-health"[All Fields] OR "electronic health"[All Fields] OR "virtual"[All Fields] OR "virtuality"[All Fields] OR "virtualization"[All Fields] OR "virtualized"[All Fields] OR "virtualizing"[All Fields] OR "virtuals"[All Fields])) | 4302 | Aug 23 2020 |
| 3 | (tw:(psychiatr* or psycholog* or "mental health" or anxiety or panic or agora* or phobia or OCD or Bipolar or "conduct disorder" or "eating disorder" or anorexia or bulimia or "mood disorder" or depress* or dysthymia or affect or psycho* or neurotic or paraphilia or schizo* or autism or ADHD or trauma or PTSD or stress OR substance abuse OR "recreational drug" or "personality disorder" or counsel* OR CBT or psychotherapy or "behavioral therapy" or ECT or "electroconvulsive therapy" or psychoanalysis)) AND (tw:("tele*"[All Fields] OR "digital*"[All Fields] OR "remote*"[All Fields] OR "video*"[All Fields] OR "Ehealth"[All Fields] OR "e-health"[All Fields] OR "e-health"[All Fields] OR "electronic health"[All Fields] OR "virtual"[All Fields] OR "virtuality"[All Fields] OR "virtualization"[All Fields] OR "virtualized"[All Fields] OR "virtualizing"[All Fields] OR "virtuals"[All Fields])) | 613 | Aug 23 2020 |

## Supplementary Material 4: List of Included Studies

1. Abbass A, Elliott J. Emotion-focused and video-technology considerations in the COVID-19 crisis. Couns Psychol Q. 2020.

2. Aboujaoude E, Gega L, Parish MB, Hilty DM. Editorial: Digital Interventions in Mental Health: Current Status and Future Directions. Frontiers in psychiatry. 2020;11:111-.

3. Alavi Z, Haque R, Felzer-Kim IT, Lewicki T, Haque A, Mormann M. Implementing COVID-19 Mitigation in the Community Mental Health Setting: March 2020 and Lessons Learned. Community Ment Health J. 2020:1-7.

4. Alexopoulos AR, Hudson JG, Otenigbagbe O. The Use of Digital Applications and COVID-19. Community Ment Health J. 2020;56(7):1202-3.

5. Ameis SH, Lai MC, Mulsant BH, Szatmari P. Coping, fostering resilience, and driving care innovation for autistic people and their families during the COVID-19 pandemic and beyond. Mol Autism. 2020;11(1):61.

6. Anton MT, Ridings LE, Gavrilova Y, Bravoco O, Ruggiero KJ, Davidson TM. Transitioning a technology-assisted stepped-care model for traumatic injury patients to a fully remote model in the age of COVID-19. Couns Psychol Q. 2020.

7. Balcombe L, De Leo D. An Integrated Blueprint for Digital Mental Health Services Amidst COVID-19. JMIR Ment Health. 2020;7(7):e21718.

8. Banducci AN, Weiss NH. Caring for patients with posttraumatic stress and substance use disorders during the COVID-19 pandemic. Psychol Trauma. 2020;12:S113-S4.

9. Barney A, Buckelew S, Mesheriakova V, Raymond-Flesch M. The COVID-19 Pandemic and Rapid Implementation of Adolescent and Young Adult Telemedicine: Challenges and Opportunities for Innovation. Journal of Adolescent Health. 2020;67(2):164-71.

10. Bate J, Malberg N. Containing the Anxieties of Children, Parents and Families from a Distance During the Coronavirus Pandemic. J Contemp Psychother. 2020:1-10.

11. Békés V, Aafjes-van Doorn K, Prout TA, Hoffman L. Stretching the Analytic Frame: Analytic Therapists’ Experiences with Remote Therapy During COVID-19. J Am Psychoanal Assoc. 2020;68(3):437-46.

12. Ben-Zeev D, Buck B, Meller S, Hudenko WJ, Hallgren KA. Augmenting Evidence-Based Care With a Texting Mobile Interventionist: A Pilot Randomized Controlled Trial. Psychiatr Serv. 2020:appips202000239.

13. Benecke AV, Bauerle A, Jansen C, Schneider JS, Dorrie N, Teufel M, et al. Techniques, Methods, and Dissemination of Community-Based Psychological Support Strategies in Times of the COVID-19 Pandemic. J Prim Care Community Health. 2020;11:2150132720943328.

14. Bennett CB, Ruggero CJ, Sever AC, Yanouri L. eHealth to redress psychotherapy access barriers both new and old: A review of reviews and meta-analyses. J Psychother Integr. 2020;2(30):188-207.

15. Bilder RM, Postal KS, Barisa M, Aase DM, Cullum CM, Gillaspy SR, et al. Inter Organizational Practice Committee Recommendations/Guidance for Teleneuropsychology in Response to the COVID-19 Pandemicdagger. Arch Clin Neuropsychol. 2020;35(6):647-59.

16. Blake H, Bermingham F, Johnson G, Tabner A. Mitigating the Psychological Impact of COVID-19 on Healthcare Workers: A Digital Learning Package. Int J Environ Res Public Health. 2020;17(9).

17. Bocher R, Jansen C, Gayet P, Gorwood P, Laprévote V. Réactivité et pérennité des soins psychiatriques en France à lépreuve du COVID-19. Encephale. 2020;46(3):S81-S4.

18. Boelen PA, Eisma MC, Smid GE, Keijser Jd, Lenferink LIM. Remotely Delivered Cognitive Behavior Therapy for Disturbed Grief During the COVID-19 Crisis: Challenges and Opportunities. J Loss Trauma. 2020.

19. Burgess C, Miller CJ, Franz A, Abel EA, Gyulai L, Osser D, et al. Practical lessons learned for assessing and treating bipolar disorder via telehealth modalities during the COVID-19 pandemic. Bipolar Disord. 2020.

20. Canady VA. Psychiatrists, patients favor telemedicine; challenges exist. Mental Health Weekly. 2020.

21. Canady VA. Virtual clinic to assist patients with urgent psychiatric care. Mental Health Weekly. 2020.

22. Caver KA, Shearer EM, Burks DJ, Perry K, De Paul NF, McGinn MM, et al. Telemental health training in the Veterans Administration Puget Sound Health Care System. Journal of Clinical Psychology. 2020;76(6):1108-24.

23. Chang BP, Kessler RC, Pincus HA, Nock MK. Digital approaches for mental health in the age of covid-19. BMJ. 2020;369:m2541.

24. Chen JA, Chung WJ, Young SK, Tuttle MC, Collins MB, Darghouth SL, et al. COVID-19 and telepsychiatry: Early outpatient experiences and implications for the future. Gen Hosp Psychiatry. 2020;66:89-95.

25. Chenneville T, Schwartz-Mette R. Ethical considerations for psychologists in the time of COVID-19. Am Psychol. 2020;75(5):644-54.

26. Chherawala N, Gill S. Up-to-date review of psychotherapy via videoconference: implications and recommendations for the RANZCP Psychotherapy Written Case during the COVID-19 pandemic. Australas Psychiatry. 2020:1039856220939495.

27. Childs AW, Unger A, Li L. Rapid design and deployment of intensive outpatient group-based psychiatric care using telehealth during COVID-19. J Am Med Inform Assoc. 2020.

28. Chin HP, Palchik G. Telepsychiatry in the Age of COVID: Some Ethical Considerations. Camb Q Healthc Ethics. 2020:1-5.

29. Colle R, Ait Tayeb AEK, de Larminat D, Commery L, Boniface B, Lasica PA, et al. Short-term acceptability by patients and psychiatrists of the turn to psychiatric teleconsultation in the context of the COVID-19 pandemic. Psychiatry Clin Neurosci. 2020;74(8):443-4.

30. Corruble E. A Viewpoint From Paris on the COVID-19 Pandemic: A Necessary Turn to Telepsychiatry. J Clin Psychiatry. 2020;81(3).

31. Cosgrove L, Karter JM, Morrill Z, McGinley M. Psychology and Surveillance Capitalism: The Risk of Pushing Mental Health Apps During the COVID-19 Pandemic. J Hum Psychol. 2020;5(60):611-25.

32. Cosic K, Popovic S, Sarlija M, Kesedzic I. Impact of Human Disasters and COVID-19 Pandemic on Mental Health: Potential of Digital Psychiatry. Psychiatr Danub. 2020;32(1):25-31.

33. Cowan A, Johnson R, Close H. Telepsychiatry in Psychotherapy Practice. Innov Clin Neurosci. 2020;17(4-6):23-6.

34. Crowe M, Inder M, Farmar R, Carlyle D. Delivering psychotherapy by video conference in the time of COVID-19: Some considerations. J psychiatr ment health nurs. 2020.

35. Danilewitz M, Ainsworth NJ, Bahji A, Chan P, Rabheru K. Virtual psychiatric care for older adults in the age of COVID-19: challenges and opportunities. Int j geriatr psychiatry. 2020.

36. Datta N, Derenne J, Sanders M, Lock JD. Telehealth transition in a comprehensive care unit for eating disorders: Challenges and long-term benefits. Int J Eat Disord. 2020.

37. Davis C, Ng KC, Oh JY, Baeg A, Rajasegaran K, Chew CSE. Caring for Children and Adolescents With Eating Disorders in the Current Coronavirus 19 Pandemic: A Singapore Perspective. J Adolesc Health. 2020;67(1):131-4.

38. de Brouwer H, Faure K, Goze T. [Institutional Psychotherapy during confinement situation: Adaptation of the therapeutic setting in a day hospital. Towards a virtual mental institution]. Ann Med Psychol (Paris). 2020.

39. de Girolamo G, Cerveri G, Clerici M, Monzani E, Spinogatti F, Starace F, et al. Mental Health in the Coronavirus Disease 2019 Emergency-The Italian Response. JAMA Psychiatry. 2020.

40. De Luca R, Calabro RS. How the COVID-19 pandemic is changing mental health disease management: The growing need of telecounseling in Italy. Innovations in Clinical Neuroscience. 2020;17(4):16-7.

41. DeLuca JS, Andorko ND, Chibani D, Jay SY, Rakhshan Rouhakhtar PJ, Petti E, et al. Telepsychotherapy with youth at clinical high risk for psychosis: Clinical issues and best practices during the COVID-19 pandemic. Journal of Psychotherapy Integration. 2020;30(2):304-31.

42. Dolev-Amit T, Leibovich L, Zilcha-Mano S. Repairing alliance ruptures using supportive techniques in telepsychotherapy during the COVID-19 pandemic. Couns Psychol Q. 2020.

43. Dursun OB, Turan B, Pakyurek M, Tekin A. Integrating Telepsychiatric Services into the Conventional Systems for Psychiatric Support to Health Care Workers and Their Children During COVID-19 Pandemics: Results from A National Experience. Telemed J E Health. 2020.

44. El Morr C, Ritvo P, Ahmad F, Moineddin R, Team MVC. Effectiveness of an 8-Week Web-Based Mindfulness Virtual Community Intervention for University Students on Symptoms of Stress, Anxiety, and Depression: Randomized Controlled Trial. JMIR Ment Health. 2020;7(7):e18595.

45. Endale T, St Jean N, Birman D. COVID-19 and refugee and immigrant youth: A community-based mental health perspective. Psychol Trauma. 2020;12(S1):S225-S7.

46. Espinel Z, Shultz JM. Using guidance from disaster psychiatry to frame psychiatric support for cancer patients during the COVID-19 lockdown. Psychooncology. 2020.

47. Fagiolini A, Cuomo A, Frank E. COVID-19 Diary From a Psychiatry Department in Italy. J Clin Psychiatry. 2020;81(3).

48. Feijt M, de Kort Y, Bongers I, Bierbooms J, Westerink J, W IJ. Mental Health Care Goes Online: Practitioners' Experiences of Providing Mental Health Care During the COVID-19 Pandemic. Cyberpsychol Behav Soc Netw. 2020.

49. Figueroa CA, Aguilera A. The Need for a Mental Health Technology Revolution in the COVID-19 Pandemic. Front Psychiatry. 2020;11:523.

50. Fonagy P, Campbell C, Truscott A, Fuggle P. Debate: Mentalising remotely - The AFNCCF's adaptations to the coronavirus crisis. Child Adolesc Ment Health. 2020;25(3):178-9.

51. Franchini L, Ragone N, Seghi F, Barbini B, Colombo C. Mental health services for mood disorder outpatients in Milan during COVID-19 outbreak: The experience of the health care providers at San Raffaele hospital. Psychiatry Res. 2020;292:113317.

52. Gaebel W, Stricker J. E-mental health options in the COVID-19 pandemic and beyond. Psychiatry Clin Neurosci. 2020;74(8):441-2.

53. Galea-Singer S, Newcombe D, Farnsworth-Grodd V, Sheridan J, Adams P, Walker N. Challenges of virtual talking therapies for substance misuse in New Zealand during the COVID-19 pandemic: an opinion piece. The New Zealand medical journal. 2020;133(1515):104-11.

54. Gautam M, Thakrar A, Akinyemi E, Mahr G. Current and Future Challenges in the Delivery of Mental Healthcare during COVID-19. SN Compr Clin Med. 2020;2(7):1-6.

55. Gentile A, De Berardis D, Tomasetti C, Ventriglio A. Digital psychiatry in COVID-19 pandemic: An Italian perspective. Psychiatry Res. 2020;292:113316.

56. Geoffroy PA, Le Goanvic V, Sabbagh O, Richoux C, Weinstein A, Dufayet G, et al. Psychological Support System for Hospital Workers During the Covid-19 Outbreak: Rapid Design and Implementation of the Covid-Psy Hotline. Front Psychiatry. 2020;11:511.

57. Giordano F, Scarlata E, Baroni M, Gentile E, Puntillo F, Brienza N, et al. Receptive music therapy to reduce stress and improve wellbeing in Italian clinical staff involved in COVID-19 pandemic: A preliminary study. Arts Psychother. 2020;70(70):101688.

58. Golberstein E, Wen H, Miller BF. Coronavirus Disease 2019 (COVID-19) and Mental Health for Children and Adolescents. JAMA Pediatrics. 2020;174(9):819-20.

59. Goodman-Casanova JM, Dura-Perez E, Guzman-Parra J, Cuesta-Vargas A, Mayoral-Cleries F. Telehealth Home Support During COVID-19 Confinement for Community-Dwelling Older Adults With Mild Cognitive Impairment or Mild Dementia: Survey Study. J Med Internet Res. 2020;22(5):e19434.

60. Gould CE, Hantke NC. Promoting Technology and Virtual Visits to Improve Older Adult Mental Health in the Face of COVID-19. Am J Geriatr Psychiatry. 2020;28(8):889-90.

61. Graell M, Moron-Nozaleda MG, Camarneiro R, Villasenor A, Yanez S, Munoz R, et al. Children and adolescents with eating disorders during COVID-19 confinement: Difficulties and future challenges. Eur Eat Disord Rev. 2020.

62. Gurwitch RH, Salem H, Nelson MM, Comer JS. Leveraging parent-child interaction therapy and telehealth capacities to address the unique needs of young children during the COVID-19 public health crisis. Psychol Trauma. 2020;12(S1):S82-S4.

63. Hames JL, Bell DJ, Perez-Lima LM, Holm-Denoma JM, Rooney T, Charles NE, et al. Navigating uncharted waters: Considerations for training clinics in the rapid transition to telepsychology and telesupervision during COVID-19. Journal of Psychotherapy Integration. 2020;30(2):348-65.

64. Haque SN. Telehealth Beyond COVID-19. Psychiatr Serv. 2020:appips202000368.

65. Harris M, Johnson S, Mackin S, Saitz R, Walley AY, Taylor JL. Low Barrier Tele-Buprenorphine in the Time of COVID-19: A Case Report. J Addict Med. 2020;14(4):e136-e8.

66. Hau YS, Kim JK, Hur J, Chang MC. How about actively using telemedicine during the COVID-19 pandemic? J Med Syst. 2020;44(6):108.

67. Haun MW, Hoffmann M, Tönnies J, Dinger U, Hartmann M, Friederich H-C. Realtime video consultations by psychotherapists in times of the COVID-19 pandemic: Effectiveness, mode of delivery and first experiences from a German feasibility study and with a routine service option in hospitals. Psychotherapeut. 2020.

68. Haxhihamza K, Arsova S, Bajraktarov S, Kalpak G, Stefanovski B, Novotni A, et al. Patient Satisfaction with Use of Telemedicine in University Clinic of Psychiatry: Skopje, North Macedonia During COVID-19 Pandemic. Telemed J E Health. 2020.

69. Hewitt KC, Rodgin S, Loring DW, Pritchard AE, Jacobson LA. Transitioning to telehealth neuropsychology service: Considerations across adult and pediatric care settings. The Clinical Neuropsychologist. 2020;34(7-8):1335-51.

70. Hom MA, Weiss RB, Millman ZB, Christensen K, Lewis EJ, Cho S, et al. Development of a virtual partial hospital program for an acute psychiatric population: Lessons learned and future directions for telepsychotherapy. Journal of Psychotherapy Integration. 2020;30(2):366-82.

71. Hser YI, Mooney LJ. Integrating Telemedicine for Medication Treatment for Opioid Use Disorder in Rural Primary Care: Beyond the COVID Pandemic. J Rural Health. 2020.

72. Hu N, Pan S, Sun J, Wang Z, Mao H. Mental health treatment online during the COVID-19 outbreak. Eur Arch Psychiatry Clin Neurosci. 2020;270(6):783-4.

73. Humer E, Pieh C, Kuska M, Barke A, Doering BK, Gossmann K, et al. Provision of Psychotherapy during the COVID-19 Pandemic among Czech, German and Slovak Psychotherapists. Int j environ res public health (Online). 2020;17(13).

74. Ifdil I, Fadli RP, Suranata K, Zola N, Ardi Z. Online mental health services in Indonesia during the COVID-19 outbreak. Asian journal of psychiatry. 2020;51:102153-.

75. Imperatori C, Dakanalis A, Farina B, Pallavicini F, Colmegna F, Mantovani F, et al. Global Storm of Stress-Related Psychopathological Symptoms: A Brief Overview on the Usefulness of Virtual Reality in Facing the Mental Health Impact of COVID-19. Cyberpsychol Behav Soc Netw. 2020.

76. Isaacs Russell G. Remote working during the pandemic: a Q&A with Gillian Issacs Russell: Questions from the Editor and Editorial Board of the British Journal of Psychotherapy. British Journal of Psychotherapy. 2020.

77. Jain N, Jayaram M. Comment on "Digital Mental Health and COVID-19: Using Technology Today to Accelerate the Curve on Access and Quality Tomorrow". JMIR Ment Health. 2020;7(8):e23023.

78. Jobes DA, Crumlish JA, Evans AD. The COVID-19 pandemic and treating suicidal risk: The telepsychotherapy use of CAMS. J Psychother Integr. 2020;2(30):226-37.

79. Kannarkat JT, Smith NN, McLeod-Bryant SA. Mobilization of Telepsychiatry in Response to COVID-19-Moving Toward 21&lt;sup&gt;st&lt;/sup&gt; Century Access to Care. Adm Policy Ment Health. 2020;47(4):489-91.

80. Kavoor AR, Chakravarthy K, John T. Remote consultations in the era of COVID-19 pandemic: Preliminary experience in a regional Australian public acute mental health care setting. Asian J Psychiatr. 2020;51:102074.

81. Khanna R, Forbes M. Telepsychiatry as a public health imperative: Slowing COVID-19. Aust N Z J Psychiatry. 2020;54(7):758-.

82. Kola L. Global mental health and COVID-19. Lancet Psychiatry. 2020;7(8):655-7.

83. Kozloff N, Mulsant BH, Stergiopoulos V, Voineskos AN. The COVID-19 Global Pandemic: Implications for People With Schizophrenia and Related Disorders. Schizophr Bull. 2020;46(4):752-7.

84. Krzystanek M, Matuszczyk M, Krupka-Matuszczyk I, Kozmin-Burzynska A, Segiet S, Przybylo J. Tele-visit (e-visit) during the epidemic crisis: Recommendations for conducting online visits in psychiatric care. Psychiatria. 2020;17(2):61-5.

85. Kwon C-Y, Kwak H-Y, Kim JW. Using Mind-Body Modalities via Telemedicine during the COVID-19 Crisis: Cases in the Republic of Korea. Int j environ res public health (Online). 2020;17(12).

86. Lepkowsky CM. Telehealth Reimbursement Allows Access to Mental Health Care During COVID-19. Am J Geriatr Psychiatry. 2020;28(8):898-9.

87. Liem A, Sit HF, Arjadi R, Patel AR, Elhai JD, Hall BJ. Ethical standards for telemental health must be maintained during the COVID-19 pandemic. Asian J Psychiatr. 2020;53:102218.

88. Liu S, Yang L, Zhang C, Xiang YT, Liu Z, Hu S, et al. Online mental health services in China during the COVID-19 outbreak. Lancet Psychiatry. 2020;7(4):e17-e8.

89. Loew TH, Krinninger M, Kettler C. [Inpatient psychosomatics in times of the coronavirus]. Psychotherapeut (Berl). 2020:1-6.

90. Looi JC, Pring W. Private metropolitan telepsychiatry in Australia during Covid-19: current practice and future developments. Australas Psychiatry. 2020:1039856220930675.

91. Lopez-Pelayo H, Aubin HJ, Drummond C, Dom G, Pascual F, Rehm J, et al. "The post-COVID era": challenges in the treatment of substance use disorder (SUD) after the pandemic. BMC Med. 2020;18(1):241.

92. MacMullin K, Jerry P, Cook K. Psychotherapist experiences with telepsychotherapy: Pre COVID-19 lessons for a post COVID-19 world. Journal of Psychotherapy Integration. 2020;30(2):248-64.

93. Manjunatha N, Kumar CN, Math SB. Coronavirus disease 2019 pandemic: Time to optimize the potential of telepsychiatric aftercare clinic to ensure the continuity of care. Indian J Psychiatry. 2020;62(3):320-1.

94. Marasca AR, Yates DB, Schneider AMdA, Feijó LP, eira DR. Psychological assessment online: Repercussions of the new coronavirus (covid-19) pandemic on remote practice andistance teaching. Estud Psicol. 2020(37):1-11.

95. Marasca C, De Rosa A, Fabbrocini G, Cantelli M, Patri A, Vastarella M, et al. Psychological teleconsultations in patients suffering from chronic skin diseases during the COVID-19 era: a service to improve patients' quality of life. J Dermatolog Treat. 2020:1-7.

96. Markowitz J. Virtual treatment and social distancing. Lancet Psychiatry. 2020;7(5):388-9.

97. Marra DE, Hamlet KM, Bauer RM, Bowers D. Validity of teleneuropsychology for older adults in response to COVID-19: A systematic and critical review. Clin Neuropsychol. 2020:1-42.

98. Marra DE, Hoelzle JB, Davis JJ, Schwartz ES. Initial changes in neuropsychologists clinical practice during the COVID-19 pandemic: a survey study. Clin Neuropsychol. 2020:1-16.

99. Marshall JM, Dunstan DA, Bartik W. The role of digital mental health resources to treat trauma symptoms in Australia during COVID-19. Psychol Trauma. 2020;12(S1):S269-S71.

100. Martin JN, Millán F, Campbell LF. Telepsychology practice: Primer and first steps. Practice Innovations. 2020;5(2):114-27.

101. Matheson BE, Bohon C, Lock J. Family-based treatment via videoconference: Clinical recommendations for treatment providers during COVID-19 and beyond. Int J Eat Disord. 2020;53(7):1142-54.

102. Mattar S, Piwowarczyk LA. COVID-19 and U.S.-based refugee populations: Commentary. Psychol Trauma. 2020;12(S1):S228-S9.

103. Matthewson J, Tiplady A, Gerakios F, Foley A, Murphy E. Implementation and analysis of a telephone support service during COVID-19. Occup Med (Lond). 2020;70(5):375-81.

104. McBeath AG, du Plock S, Bager-Charleson S. The challenges and experiences of psychotherapists working remotely during the coronavirus pandemic. Couns Psychother Res. 2020.

105. McGrath J. ADHD and Covid-19: current roadblocks and future opportunities. Ir J Psychol Med. 2020:1-8.

106. Medalia A, Lynch DA, Herlands T. Telehealth Conversion of Serious Mental Illness Recovery Services During the COVID-19 Crisis. Psychiatr Serv. 2020;71(8):872.

107. Merchant R, Torous J, Rodriguez-Villa E, Naslund JA. Digital technology for management of severe mental disorders in low-income and middle-income countries. Curr Opin Psychiatry. 2020;33(5):501-7.

108. Miermont-Schilton D, Richard F. The current sociosanitary coronavirus crisis: remote psychoanalysis by Skype or telephone. Int J Psychoanal. 2020;3(101):572-9.

109. Millar C, Campbell S, Fisher P, Hutton J, Morgan A, Cherry MG. Cancer and COVID-19: Patients' and psychologists' reflections regarding psycho-oncology service changes. Psychooncology. 2020.

110. Moring JC, Dondanville KA, Fina BA, Hassija C, Chard K, Monson C, et al. Cognitive Processing Therapy for Posttraumatic Stress Disorder via Telehealth: Practical Considerations During the COVID-19 Pandemic. J Trauma Stress. 2020.

111. Mosolov SN. [Problems of mental health in the situation of COVID-19 pandemic]. Zh Nevrol Psikhiatr Im S S Korsakova. 2020;120(5):7-15.

112. Murphy R, Calugi S, Cooper Z, Dalle Grave R. Challenges and Opportunities for enhanced cognitive behaviour therapy (CBT-E) in light of COVID-19. Cogn Behav Ther. 2020.

113. Naal H, Whaibeh E, Mahmoud H. Guidelines for primary health care-based telemental health in a low-to middle-income country: the case of Lebanon. Int Rev Psychiatry. 2020:1-9.

114. Naarding P, Oude Voshaar RC, Marijnissen RM. COVID-19: Clinical Challenges in Dutch Geriatric Psychiatry. The American Journal of Geriatric Psychiatry. 2020;28(8):839-43.

115. Nagata JM. Rapid Scale-Up of Telehealth During the COVID-19 Pandemic and Implications for Subspecialty Care in Rural Areas. J Rural Health. 2020.

116. Nahanadi MZ, Shahrokhi H, Farhang S, Somi MH. Virtual social networks and mental health intervention for medical staff during the COVID-19 outbreak in the Islamic Republic of Iran. East Mediterr Health J. 2020;26(5):497-8.

117. Narzisi A. Phase 2 and Later of COVID-19 Lockdown: Is it Possible to Perform Remote Diagnosis and Intervention for Autism Spectrum Disorder? An Online-Mediated Approach. Journal of clinical medicine. 2020.

118. O'Brien M, McNicholas F. The use of telepsychiatry during COVID-19 and beyond. Ir J Psychol Med. 2020:1-6.

119. Ojha R, Syed S. Challenges faced by mental health providers and patients during the coronavirus 2019 pandemic due to technological barriers. Internet Interventions. 2020;21:100330.

120. Olwill C, Mc Nally D, Douglas L. Psychiatrist experience of remote consultations by telephone in an outpatient psychiatric department during the COVID-19 pandemic. Ir J Psychol Med. 2020:1-8.

121. Patel S, Gannon A, Dolan C, McCarthy G. Telehealth in Psychiatry of Old Age: Ordinary Care in Extraordinary Times in Rural North-West Ireland. Am J Geriatr Psychiatry. 2020;28(9):1009-11.

122. Payne L, Flannery H, Kambakara Gedara C, Daniilidi X, Hitchcock M, Lambert D, et al. Business as usual? Psychological support at a distance. Clin Child Psychol Psychiatry. 2020;25(3):672-86.

123. Pelizza L, Pupo S. Public mental health outpatient service at the time of the COVID-19 pandemic: Did we have any other choice? Journal of Psychopathology. 2020;26(2):186-7.

124. Perrin PB, Rybarczyk BD, Pierce BS, Jones HA, Shaffer C, Islam L. Rapid telepsychology deployment during the COVID-19 pandemic: A special issue commentary and lessons from primary care psychology training. J Clin Psychol. 2020;76(6):1173-85.

125. Peterson RK, Ludwig NN, Jashar DT. A case series illustrating the implementation of a novel tele-neuropsychology service model during COVID-19 for children with complex medical and neurodevelopmental conditions: A companion to Pritchard et al., 2020. Clin Neuropsychol. 2020:1-16.

126. Pierce BS, Perrin PB, Tyler CM, McKee GB, Watson JD. The COVID-19 telepsychology revolution: A national study of pandemic-based changes in U.S. mental health care delivery. Am Psychol. 2020.

127. Ping NPT, Shoesmith WD, James S, Nor Hadi NM, Yau EKB, Lin LJ. Ultra Brief Psychological Interventions for COVID-19 Pandemic: Introduction of a Locally-Adapted Brief Intervention for Mental Health and Psychosocial Support Service. Malays J Med Sci. 2020;27(2):51-6.

128. Pinto da Costa M. Can social isolation caused by physical distance in people with psychosis be overcome through a Phone Pal? Eur Psychiatry. 2020;63(1):e61-e.

129. Pritchard AE, Sweeney K, Salorio CF, Jacobson LA. Pediatric neuropsychological evaluation via telehealth: Novel models of care. Clin Neuropsychol. 2020:1-13.

130. Probst T, Stippl P, Pieh C. Changes in Provision of Psychotherapy in the Early Weeks of the COVID-19 Lockdown in Austria. Int J Environ Res Public Health. 2020;17(11).

131. Ragavan MI, Culyba AJ, Muhammad FL, Miller E. Supporting Adolescents and Young Adults Exposed to or Experiencing Violence During the COVID-19 Pandemic. J Adolesc Health. 2020;67(1):18-20.

132. Ramalho R, Adiukwu F, Gashi Bytyci D, El Hayek S, Gonzalez-Diaz JM, Larnaout A, et al. Telepsychiatry and healthcare access inequities during the COVID-19 pandemic. Asian J Psychiatr. 2020;53:102234.

133. Ramtekkar U, Bridge J, Thomas G, Butter E, Reese J, Logan E, et al. Pediatric Telebehavioral Health: A Transformational Shift in Care Delivery in the Era of COVID-19. JMIR Ment Health. 2020.

134. Razai MS, Oakeshott P, Kankam H, Galea S, Stokes-Lampard H. Mitigating the psychological effects of social isolation during the covid-19 pandemic. BMJ. 2020;369:m1904.

135. Reay RE, Looi JC, Keightley P. Telehealth mental health services during COVID-19: summary of evidence and clinical practice. Australas Psychiatry. 2020:1039856220943032.

136. Riegler LJ, Raj SP, Moscato EL, Narad ME, Kincaid A, Wade SL. Pilot trial of a telepsychotherapy parenting skills intervention for veteran families: Implications for managing parenting stress during COVID-19. Journal of Psychotherapy Integration. 2020;30(2):290-303.

137. Rodriguez KA. Maintaining Treatment Integrity in the Face of Crisis: A Treatment Selection Model for Transitioning Direct ABA Services to Telehealth. Behav Anal Pract. 2020;13(2):1-8.

138. Roncero C, Garcia-Ullan L, de la Iglesia-Larrad JI, Martin C, Andres P, Ojeda A, et al. The response of the mental health network of the Salamanca area to the COVID-19 pandemic: The role of the telemedicine. Psychiatry Res. 2020;291:113252.

139. Ronen-Setter IH, Cohen E. Becoming "Teletherapeutic": Harnessing Accelerated Experiential Dynamic Psychotherapy (AEDP) for Challenges of the Covid-19 Era. J Contemp Psychother. 2020:1-9.

140. Rorai V, Perry TE. An Innovative Telephone Outreach Program to Seniors in Detroit, a City Facing Dire Consequences of COVID-19. Journal of Gerontological Social Work. 2020;63(6-7):713-6.

141. Rosen CS, Glassman LH, Morl, LA. Telepsychotherapy during a pandemic: A traumatic stress perspective. Journal of Psychotherapy Integration. 2020;30(2):174-87.

142. Rosic T, Lubert S, Samaan Z. Virtual psychiatric care fast-tracked: Reflections inspired by the COVID-19 pandemic. BJPsych Bull. 2020:1-8.

143. Salum GA, Rehmenklau JF, Csordas MC, Pereira FP, Castan JU, Ferreira AB, et al. Supporting people with severe mental health conditions during the COVID-19 pandemic: considerations for low- and middle-income countries using telehealth case management. Braz J Psychiatry. 2020;42(4):451-2.

144. Sammons MT, VandenBos GR, Martin JN. Psychological Practice and the COVID-19 Crisis: A Rapid Response Survey. J Health Serv Psychol. 2020:1-7.

145. Samuels EA, Clark SA, Wunsch C, Jordison Keeler LA, Reddy N, Vanjani R, et al. Innovation During COVID-19: Improving Addiction Treatment Access. J Addict Med. 2020;14(4):e8-e9.

146. Sansom-Daly UM, Bradford N. Grappling with the 'human' problem hiding behind the technology: Telehealth during and beyond COVID-19. Psychooncology. 2020.

147. Schieltz KM, Wacker DP. Functional assessment and function-based treatment delivered via telehealth: A brief summary. J Appl Behav Anal. 2020;53(3):1242-58.

148. Schuh Teixeira AL, Spadini AV, Pereira-Sanchez V, Ojeahere MI, Morimoto K, Chang A, et al. The urge to implement and expand telepsychiatry during the COVID-19 crisis: Early career psychiatrists' perspective. Rev Psiquiatr Salud Ment. 2020;13(3):174-5.

149. Sequeira A, Alozie A, Fasteau M, Lopez AK, Sy J, Turner KA, et al. Transitioning to virtual programming amidst COVID-19 outbreak. Couns Psychol Q. 2020.

150. Sharma A, Sasser T, Schoenfelder Gonzalez E, Vander Stoep A, Myers K. Implementation of Home-Based Telemental Health in a Large Child Psychiatry Department During the COVID-19 Crisis. J Child Adolesc Psychopharmacol. 2020.

151. Shore JH, Schneck CD, Mishkind MC. Telepsychiatry and the Coronavirus Disease 2019 Pandemic-Current and Future Outcomes of the Rapid Virtualization of Psychiatric Care. JAMA Psychiatry. 2020.

152. Sklar M, Reeder K, Carandang K, Ehrhart MG, Aarons GA. An Observational Study of the Impact of COVID-19 and the Transition to Telehealth on Community Mental Health Center Providers. Research Square [Preprint]. 2020.

153. Smith K, Ostinelli E, Macdonald O, Cipriani A. COVID-19 and Telepsychiatry: Development of Evidence-Based Guidance for Clinicians. JMIR Ment Health. 2020;7(8):e21108.

154. Sockalingam S, Clarkin C, Serhal E, Pereira C, Crawford A. Responding to Health Care Professionals' Mental Health Needs During COVID-19 Through the Rapid Implementation of Project ECHO. J Contin Educ Health Prof. 2020.

155. Sorinmade OA, Kossoff L, Peisah C. COVID-19 and Telehealth in older adult psychiatry-opportunities for now and the future. Int J Geriatr Psychiatry. 2020.

156. Soron TR, Shariful Islam SM, Ahmed HU, Ahmed SI. The hope and hype of telepsychiatry during the COVID-19 pandemic. Lancet Psychiatry. 2020;7(8):e50.

157. Sousa A, Karia S. Telepsychiatry during COVID-19: Some clinical, public health, and ethical dilemmas. Indian J Public Health. 2020;64(Supplement):S245-S6.

158. Stewart RW, Orengo-Aguayo R, Young J, Wallace MM, Cohen JA, Mannarino AP, et al. Feasibility and effectiveness of a telehealth service delivery model for treating childhood posttraumatic stress: A community-based, open pilot trial of trauma-focused cognitive-behavioral therapy. Journal of Psychotherapy Integration. 2020;30(2):274-89.

159. Stoll J, Sadler JZ, Trachsel M. The Ethical Use of Telepsychiatry in the Covid-19 Pandemic. Front Psychiatry. 2020;11:665.

160. Sullivan AB, Kane A, Roth AJ, Davis BE, Drerup ML, Heinberg LJ. The COVID-19 Crisis: A Mental Health Perspective and Response Using Telemedicine. J Patient Exp. 2020.

161. Svenson K. Teleanalytic Therapy in the Era of Covid-19: Dissociation in the Countertransference. J Am Psychoanal Assoc. 2020;68(3):447-54.

162. Taylor CB, Fitzsimmons-Craft EE, Graham AK. Digital technology can revolutionize mental health services delivery: The COVID-19 crisis as a catalyst for change. Int J Eat Disord. 2020;53(7):1155-7.

163. Thirthalli J, Manjunatha N, Math SB. Unmask the mind! Importance of video consultations in psychiatry during COVID-19 pandemic. Schizophr Res. 2020.

164. Thomas RK, Suleman R, Mackay M, Hayer L, Singh M, Correll CU, et al. Adapting to the impact of COVID-19 on mental health: an international perspective. J Psychiatry Neurosci. 2020;45(4):229-33.

165. Thome J, Coogan AN, Fischer M, Tucha O, Faltraco F. Challenges for mental health services during the 2020 COVID-19 outbreak in Germany. Psychiatry and clinical neurosciences. 2020;74(7):407-.

166. Thompson-de Benoit A, Kramer U. Work with emotions in remote psychotherapy in the time of Covid-19: a clinical experience. Couns Psychol Q. 2020.

167. Thum CC, Chai YC, Zaman Huri S, Wan Nawawi WZ, Ibrahim N. Innovative psychological first aid (PFA) in the new normal for frontliners. Perspect Psychiatr Care. 2020.

168. Titov N, Staples L, Kayrouz R, Cross S, Karin E, Ryan K, et al. Rapid report: Early demand, profiles and concerns of mental health users during the coronavirus (COVID-19) pandemic. Internet Interv. 2020;21:100327.

169. Torous J, Keshavan M. COVID-19, mobile health and serious mental illness. Schizophr Res. 2020;218:36-7.

170. Tullio V, Perrone G, Bilotta C, Lanzarone A, Argo A. Psychological support and psychotherapy via digital devices in Covid-19 emergency time: Some critical issues. Med Leg J. 2020;88(2):73-6.

171. Usman M, Fahy S. Coping with the COVID-19 crisis: an overview of service adaptation and challenges encountered by a rural Psychiatry of Later Life (POLL) team. Ir J Psychol Med. 2020:1-5.

172. Van Daele T, Karekla M, Kassianos AP, Compare A, Haddouk L, Salgado J, et al. Recommendations for policy and practice of telepsychotherapy and e-mental health in Europe and beyond. Journal of Psychotherapy Integration. 2020;30(2):160-73.

173. Van Orden KA, Bower E, Lutz J, Silva C, Gallegos AM, Podgorski CA, et al. Strategies to Promote Social Connections Among Older Adults During 'Social Distancing' Restrictions. Am J Geriatr Psychiatry. 2020.

174. Viswanathan R, Myers MF, Fanous AH. Support Groups and Individual Mental Health Care via Video Conferencing for Frontline Clinicians During the COVID-19 Pandemic. Psychosomatics. 2020.

175. Wade SL, Gies LM, Fisher AP, Moscato EL, Adlam AR, Bardoni A, et al. Telepsychotherapy with children and families: Lessons gleaned from two decades of translational research. Journal of Psychotherapy Integration. 2020;30(2):332-47.

176. Waller G, Pugh M, Mulkens S, ra, Moore E, Mountford VA, et al. Cognitive-behavioral therapy in the time of coronavirus: Clinician tips for working with eating disorders via telehealth when face-to-face meetings are not possible. Int J Eat Disord. 2020;53(7):1132-41.

177. Wang L, Fagan C, Yu CL. Popular mental health apps (MH Apps) as a complement to telepsychotherapy: Guidelines for consideration. Journal of Psychotherapy Integration. 2020;30(2):265-73.

178. Warnock-Parkes E, Wild J, Thew GR, Kerr A, Grey N, Stott R, et al. Treating Social Anxiety Disorder Remotely with Cognitive Therapy. The Cognitive Behaviour Therapist. 2020.

179. Watts S, March, A, Bouchard S, Gosselin P, Langlois F, et al. Telepsychotherapy for generalized anxiety disorder: Impact on the working alliance. Journal of Psychotherapy Integration. 2020;30(2):208-25.

180. Weissman RS, Bauer S, Thomas JJ. Access to evidence-based care for eating disorders during the COVID-19 crisis. Int J Eat Disord. 2020;53(5):369-76.

181. Wells SY, Morland LA, Wilhite ER, Grubbs KM, Rauch SAM, Acierno R, et al. Delivering Prolonged Exposure Therapy via Videoconferencing During the COVID-19 Pandemic: An Overview of the Research and Special Considerations for Providers. J Trauma Stress. 2020.

182. Whaibeh E, Mahmoud H, Naal H. Telemental Health in the Context of a Pandemic: the COVID-19 Experience. Curr Treat Options Psychiatry. 2020:1-5.

183. Whelan P, Stockton-Powdrell C, Jardine J, Sainsbury J. Comment on "Digital Mental Health and COVID-19: Using Technology Today to Accelerate the Curve on Access and Quality Tomorrow": A UK Perspective. JMIR Ment Health. 2020;7(4):e19547.

184. Wind TR, Rijkeboer M, Andersson G, Riper H. The COVID-19 pandemic: The 'black swan' for mental health care and a turning point for e-health. Internet Interv. 2020;20:100317.

185. Wood SM, White K, Peebles R, Pickel J, Alausa M, Mehringer J, et al. Outcomes of a Rapid Adolescent Telehealth Scale-Up During the COVID-19 Pandemic. J Adolesc Health. 2020;67(2):172-8.

186. Wright JH, Caudill R. Remote Treatment Delivery in Response to the COVID-19 Pandemic. Psychother Psychosom. 2020;89(3):130-2.

187. Xiang YT, Zhao N, Zhao YJ, Liu Z, Zhang Q, Feng Y, et al. An overview of the expert consensus on the mental health treatment and services for major psychiatric disorders during COVID-19 outbreak: China's experiences. Int J Biol Sci. 2020;16(13):2265-70.

188. Yao H, Chen J-H, Xu Y-F. Rethinking online mental health services in China during the COVID-19 epidemic. Asian journal of psychiatry. 2020;50:102015-.

189. Yellowlees P, Nakagawa K, Pakyurek M, Hanson A, Elder J, Kales HC. Rapid Conversion of an Outpatient Psychiatric Clinic to a 100% Virtual Telepsychiatry Clinic in Response to COVID-19. Psychiatr Serv. 2020;71(7):749-52.

190. Zarghami A, Farjam M, Fakhraei B, Hashemzadeh K, Yazdanpanah MH. A Report of the Telepsychiatric Evaluation of SARS-CoV-2 Patients. Telemed J E Health. 2020.

191. Zhai Y. A Call for Addressing Barriers to Telemedicine: Health Disparities during the COVID-19 Pandemic. Psychother Psychosom. 2020:1-3.

192. Zhang C, Zhu K, Li D, Voon V, Sun B. Deep brain stimulation telemedicine for psychiatric patients during the COVID-19 pandemic. Brain Stimul. 2020;13(5):1263-4.

193. Zhang M, Smith HE. Digital Tools to Ameliorate Psychological Symptoms Associated With COVID-19: Scoping Review. J Med Internet Res. 2020;22(8):e19706.

194. Zhou J, Liu L, Xue P, Yang X, Tang X. Mental Health Response to the COVID-19 Outbreak in China. Am J Psychiatry. 2020;177(7):574-5.

195. Zhou X, Snoswell CL, Harding LE, Bambling M, Edirippulige S, Bai X, et al. The Role of Telehealth in Reducing the Mental Health Burden from COVID-19. Telemed J E Health. 2020;26(4):377-9.

196. Zulfic Z, Liu D, Lloyd C, Rowan J, Schubert KO. Is telepsychiatry care a realistic option for community mental health services during the COVID-19 pandemic? Aust N Z J Psychiatry. 2020:4867420937788.

## Supplementary Material 5:

## World Bank Classification of Countries

**Income Group Country Number Frequency**

Low Income Countries n/a n/a n/a

Lower-Middle Income Countries

India 3.00 0.02

Bangladesh 1.00 0.01

Upper-Middle Income Countries

China 4.00 0.02

Brazil 2.00 0.01

Iran 2.00 0.01

Malaysia 2.00 0.01

Indonesia 1.00 0.01

Lebanon 1.00 0.01

Macedonia 1.00 0.01

Russia 1.00 0.01

Turkey 1.00 0.01

High Income Countries

USA 31.00 0.16

Italy 6.00 0.03

UK 6.00 0.03

Canada 5.00 0.03

France 5.00 0.03

Ireland 4.00 0.02

Australia 4.00 0.02

Germany 3.00 0.02

Austria 1.00 0.01

Croatia 1.00 0.01 Netherlands 1.00 0.01

New Zealand 1.00 0.01

Singapore 1.00 0.01

Spain 1.00 0.01

Switzerland 1.00 0.01

Multiple Countries 12.00 0.06

Unspecified 95.00 0.48

## World Health Organization Regional Classification of countries

**Region Country Number Frequency**

Africa n/a n/a n/a

Americas

USA 31.00 0.16

Canada 5.00 0.03

Brazil 2.00 0.01

Eastern Mediterranean

Iran 2.00 0.01

Lebanon 1.00 0.01

Europe

Italy 6.00 0.03

UK 6.00 0.03

France 5.00 0.03

Ireland 4.00 0.02

Germany 3.00 0.02

Austria 1.00 0.01

Croatia 1.00 0.01

Macedonia 1.00 0.01 Netherlands 1.00 0.01 Russia 1.00 0.01

Spain 1.00 0.01 Switzerland 1.00 0.01 Turkey 1.00 0.01

South East Asia

India 3.00 0.02

Bangladesh 1.00 0.01

Indonesia 1.00 0.01

Western Pacific

Australia 4.00 0.02

China 4.00 0.02

Malaysia 2.00 0.01

New Zealand 1.00 0.01

Singapore 1.00 0.01

Multiple Countries 12.00 0.06

Unspecified 95.00 0.48

## Supplementary Material 6: Healthcare Providers

1. Addiction specialists
2. Behavior analysts
3. Behavior therapists
4. Clinicians/Doctors/Physicians
5. Community Center harm reduction specialists
6. Counselors
7. Healthcare professionals/practitioners
8. Mental health professionals/providers
9. Neuropsychologists
10. Nurses and nurse practitioners
11. Psychiatrists
12. Psychoanalysts
13. Psychologists
14. Psychometrists
15. Social workers
16. Therapists
17. Volunteers

## Supplementary Material 7: Tele-mental Health Service Technique

1. At-home Deep Brain Stimulation (DBS)
2. Computer-assisted cognitive-behavioral therapy (CCBT)
3. Cognitive Behavior Therapy (CBT) via telehealth
4. Cognitive Processing Therapy (CPT) via Telehealth
5. Cognitive-behavior framework via telephones
6. Digital mental health
7. Digital mindfulness and Cognitive Behavioral Therapy
8. Digital psychiatry
9. e-mental health
10. e-psychotherapy
11. Family-based treatment (FBT) via tele-health
12. Internet-delivered cognitive behavioral therapy (i-CBT)
13. Mentalization-based treatment for children via tele-therapy
14. Prolonged exposure therapy (PE) via video teleconferencing
15. Psychiatric teleconsultation
16. Psychiatry via m-health
17. Psychiatry via tele-medicine
18. Remote analytic therapy
19. Remote Cognitive Behavioral Therapy
20. Remote Family Based Treatment (FBT)
21. Remote Cognitive Remediation (CRT)
22. Remote enhanced Cognitive Behavioral Therapy [CBT-E] (talking therapy)
23. Remote musical therapy
24. Remote psycho-analysis
25. Remote psychotherapy
26. Remote virtual reality-based therapy
27. Tele-analysis
28. Tele-behavioral health
29. Tele-bridge clinic (audio-only telehealth)
30. Tele-buprenorphine
31. Tele-counseling
32. Tele-mental health
33. Tele-neuropsychology
34. Tele-neuropsychology services
35. Tele-psychiatry
36. Tele-psychoanalysis
37. Tele-psychodiagnosis
38. Tele-psychology
39. Tele-psychotherapy
40. Tele-psychotherapy utilizing Core Conflictual Relationship Theme Framework
41. Tele-psychotherapy utilizing problem-solving and parent-training interventions
42. Tele-therapy
43. Virtual acceptance and commitment therapy
44. Virtual analytic therapy
45. Virtual behavioral activation
46. Virtual behavior-analytic service
47. Virtual cognitive-behavioral therapy
48. Virtual dialectal behavioral therapy
49. Virtual Cognitive-Behavioral Therapy
50. Virtual cognitive remediation
51. Virtual counselling and group therapy
52. Virtual emotions-focused therapy
53. Virtual exposure therapy
54. Virtual group psychotherapy
55. Virtual integrative, person-centered, transactional analysis
56. Virtual Interpersonal and Social Rhythm Therapy (IPSRT)
57. Virtual Interpersonal Psychotherapy (IPT)
58. Virtual mindfulness
59. Virtual Parent–Child Interaction Therapy (PCIT)
60. Virtual psychiatric care (e-Visits)
61. Virtual psycho-dynamic, existential, gestalt, humanistic, pluralist and psycho-analytic therapy
62. Virtual psychological assessment
63. Virtual psychotherapy
64. Virtual talking therapy and motivational interviewing
